# Supramolecular trap for a transient corannulene trianion†
†Electronic supplementary information (ESI) available: Details of preparation, characterization, X-ray diffraction study, and theoretical calculations. CCDC 948063. For ESI and crystallographic data in CIF or other electronic format see DOI: 10.1039/c5sc04385a


**DOI:** 10.1039/c5sc04385a

**Published:** 2015-12-17

**Authors:** Alexander V. Zabula, Sarah N. Spisak, Alexander S. Filatov, Andrey Yu. Rogachev, Rodolphe Clérac, Marina A. Petrukhina

**Affiliations:** a Department of Chemistry , University at Albany , State University of New York , Albany , NY 12222 , USA . Email: mpetrukhina@albany.edu; b Department of Chemistry , University of Pennsylvania , Philadelphia , PA 19104 , USA; c Department of Chemistry , Illinois Institute of Technology , Chicago , IL 60616 , USA . Email: arogache@iit.edu; d CNRS , Centre de Recherche Paul Pascal (CRPP) , Pessac , F-33600 , France; e Université de Bordeaux , Pessac , F-33600 , France

## Abstract

The first structural characterization of the transient triply-reduced state of corannulene (C_20_H_10_) is accomplished. The X-ray crystallographic study reveals that the C_20_H_10_˙^3–^ trianions, generated by corannulene reduction with metallic cesium, form a novel type of supramolecular sandwich-type assembly, [Cs^+^//(C_20_H_10_^3–^)/4Cs^+^/(C_20_H_10_^3–^)//Cs^+^]. In the product, two triply-charged corannulene decks encapsulate a rectangle of four cesium ions with the external concave bowl cavities being filled by one cesium ion each. The structural investigation is augmented by in-depth theoretical calculations to provide insights into the geometrical features and electronic structure of this unique organometallic self-assembly.

## Abstract 2

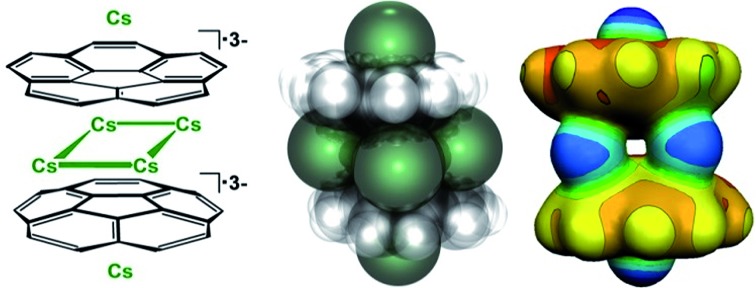
The first X-ray structural characterization of the triply-reduced corannulene (C_20_H_10_) reveals its ability to form a novel type of supramolecular assembly with large cesium ions, [Cs^+^//(C_20_H_10_^3–^)/4Cs^+^/(C_20_H_10_^3–^)//Cs^+^].

## Introduction

Multi-electron reduction of planar and non-planar polyaromatic hydrocarbons (PAHs), structures of the resulting carbanions as well as their supramolecular aggregation with alkali metal ions have been the focus of great attention in the last two decades.^1^–^3^ The discoveries of curved and bent π-conjugated molecules, such as fragments of fullerenes and nanotubes,^4^ further reinvigorated this field with the focus on aromaticity, reactivity, magnetism and electronic properties of the resulting charged carbon-rich species.^5^–^7^ Special interest in non-planar radicals with extended π-surfaces^8^ has arisen from their charge transport abilities,^9^ magnetic properties,^10^ interesting coupling pathways^11^ as well as prospective applications in organic microelectronics and energy storage.^12^ Considering a broad family of polycyclic hydrocarbons, monoanion-radicals are well-documented and have been characterized both in solution and solid state for a great number of polyaromatic scaffolds.^13^,^14^ In contrast, the number of isolated and structurally characterized trianion-radicals is limited. Only for C_60_-fullerene, the trianions are well-known,^15^ stemming from the triply degenerate nature of its low-lying unoccupied molecular orbital (LUMO) that can accept up to six electrons.^16^ For the extended family of PAHs, the trianions have been isolated and crystallographically characterized for large bisanthryl^17^ and decacyclene^18^ only. Overall, the controlled preparation of trianion-radicals of PAHs, which are often transient species on the way to more highly reduced carbanions, is challenging, while their electronic structures, properties, stability, and reactivity are especially intriguing.

**Scheme 1:**
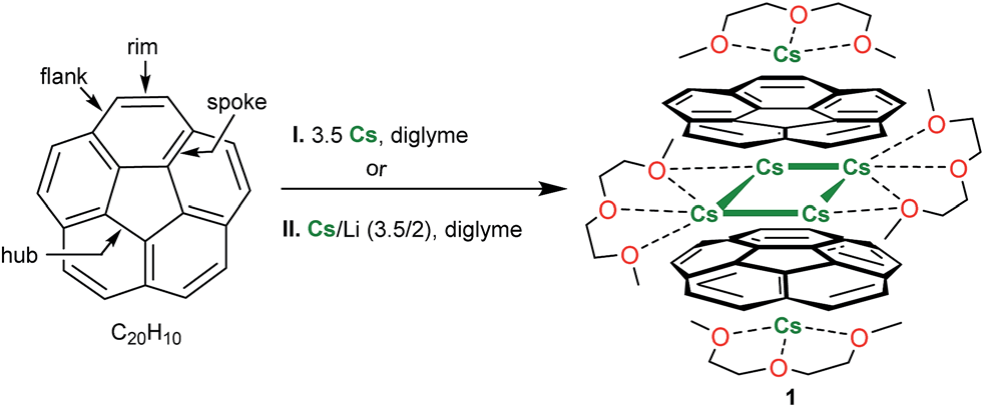
Preparation of **1**.

Bowl-shaped polyaromatic hydrocarbons, representing curved fragments of fullerenes, are known to readily uptake multiple electrons. For example, corannulene (C_20_H_10_, Scheme 1), which maps a 1/3 of the C_60_-fullerene surface, can acquire up to four electrons, owing to the doubly degenerate nature of its LUMO.^19^ For the family of corannulene anions, products of mono-,^20^ di-,20*b* and highly reduced tetraanions^21^ (C_20_H_10_^*n*–^, *n* = 1, 2, 4) have been recently isolated with different alkali metal counterions and characterized by X-ray crystallography. This allowed us to follow structural perturbations of the corannulene core upon addition of one, two, and four electrons. These studies also revealed a tendency of very electron-rich tetrareduced corannulene to form remarkable supramolecular products with the high number of encapsulated alkali metal ions, including unprecedented heterobimetallic combinations.^22^ This behavior clearly differentiates the corannulene tetraanion, C_20_H_10_^4–^, bearing the record charge per carbon atom (one electron per five C-atoms *vs.* one electron per ten C-atoms in the C_60_-fullerene hexaanion, for comparison) from mono- and doubly-charged corannulene, which can be isolated in their “naked” forms.^23^ Notably, until now the triply-reduced corannulene remained missing in the above series of structurally characterized corannulene anions.

Previously, the paramagnetic C_20_H_10_˙^3–^ radical was detected by ESR spectroscopy using the *in situ* reduction reaction of corannulene with lithium metal in THF.^24^ The ESR data provided the only evidence of existence of this transient species in solution, as the initially reported UV-vis spectroscopic data for the C_20_H_10_˙^3–^ anion24*a* have been later re-assigned to the tetrareduced state of corannulene.^25^ The question whether the corannulene trianion engages in self-assembly with alkali metal ions or can exist in discrete form has remained open. Herein, we prove that the transient C_20_H_10_˙^3–^ anion can be isolated in the solid state in the form of its cesium salt. We also reveal a remarkable supramolecular structure formed by C_20_H_10_˙^3–^ anions with multiple cesium counterions based on single crystal X-ray diffraction study and provide full characterization of this novel organometallic product. Moreover, comprehensive theoretical evaluation of unique geometric and electronic features of the new sandwich-type assembly has been accomplished.

## Results and discussion

Corannulene reacts with cesium metal (3.5 equiv.) in diglyme with the initial formation of an intense green solution characteristic of the monoanion, C_20_H_10_˙^–^.^20^ The subsequent fast reduction of the monoanion leads to the appearance of the bright purple dianion in the reaction mixture.^26^ The prolonged reaction time (up to 60 h) at ambient conditions resulted in the formation of the red-purple solution attributed to the C_20_H_10_˙^3–^ trianion. The UV-vis spectrum of this reaction mixture exhibits a very broad absorbance band around 500 nm and a very intense band, *λ*_max_ = 388 nm, characteristic of the C_20_H_10_˙^3–^ anion (ESI, Fig. S2 and S3†). The latter absorbance maximum is hypsochromically shifted compared to the most intense band of C_20_H_10_^4–^ (*λ*_max_ = 460 nm in diglyme21*b* and 429 nm in THF21*a*). Notably, no further reduction of C_20_H_10_˙^3–^ to the C_20_H_10_^4–^ state was observed in this work even when an excess of metallic cesium was used over a very extended reaction time period (more than 2 months).

We found that the target carbanion was very difficult to isolate in the crystalline form. Multiple initial attempts to crystallize corannulene trianion from the above systems led to the precipitation of oily or amorphous powders. Ultimately, we observed that the addition of dicyclohexano-18-crown-6 to the reaction mixture followed by its layering with hexanes resulted in the formation of several dark-red crystals of [Cs^+^_3_(diglyme)_2_(C_20_H_10_^3–^)] (**1**) (Scheme 1). Surprisingly, crown ether is not incorporated into the final product although its presence in the reaction solution somehow facilitated crystallization. We later found that the same crystalline product **1** can also be obtained when a mixture of cesium and lithium metals (in a 3.5 : 2 ratio with respect to corannulene) is used for the reduction reactions. Notably, the resulting crystals are very difficult to handle due to their extreme air- and moisture-sensitivity. Crystals of **1** are soluble in diglyme only and have very limited solubility in THF.

**Fig. 1:**
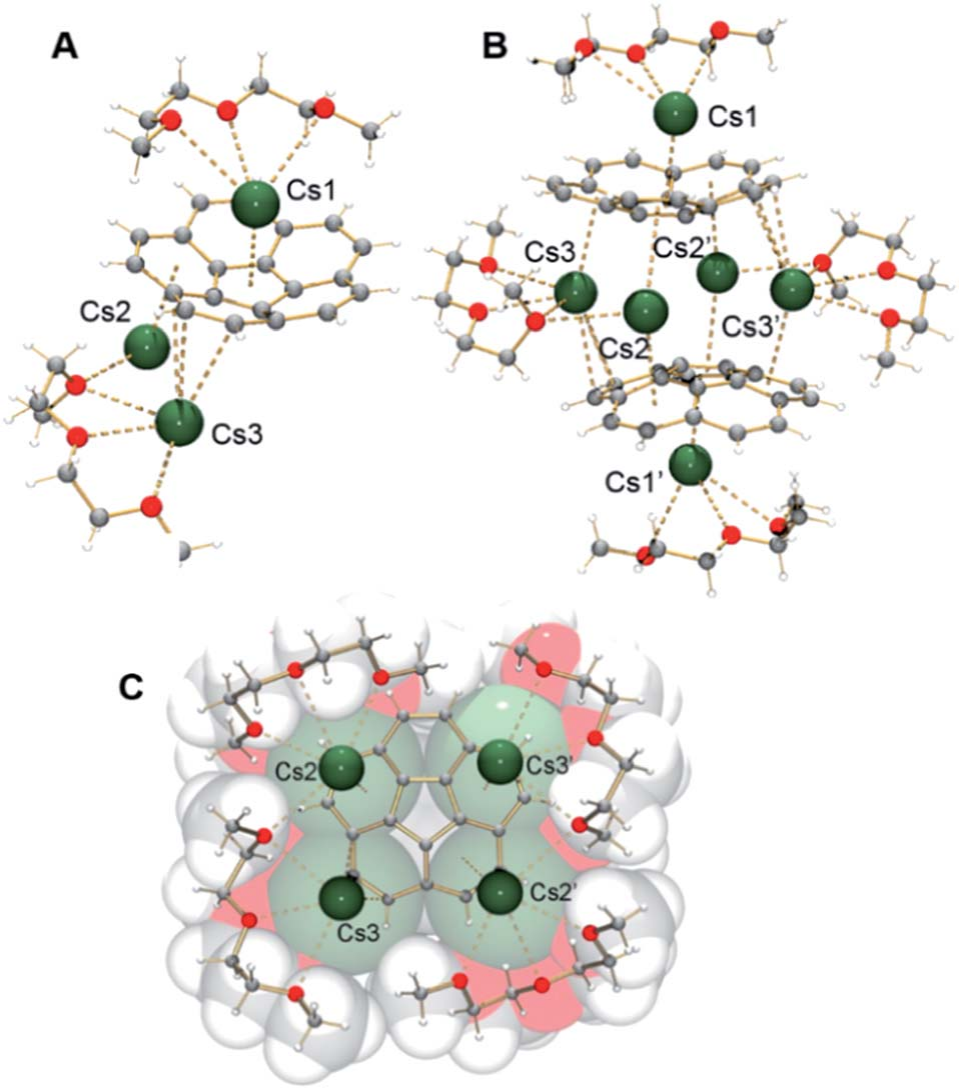
Asymmetric unit (A), sandwich view (B) and depiction of the solvated Cs^+^ ions within the sandwich, superimposed with the space filling model (C) for **1**.

The X-ray crystallographic study revealed that **1** crystallizes in the *C*2/*c* space group with the asymmetric unit consisting of one corannulene trianion, three cesium ions, and two diglyme molecules (Fig. 1A, see ESI† for more details). In the molecular structure residing on the inversion center, four cesium ions are sandwiched between two triply-reduced corannulene bowls to yield a novel type of supramolecular assembly, [(C_20_H_10_^3–^)/4Cs^+^/(C_20_H_10_^3–^)]^2–^ (Fig. 1B). A similar arrangement of four cesium ions between two tetrareduced corannulene moieties has been suggested by theoretical calculations.^27^

**Fig. 2:**
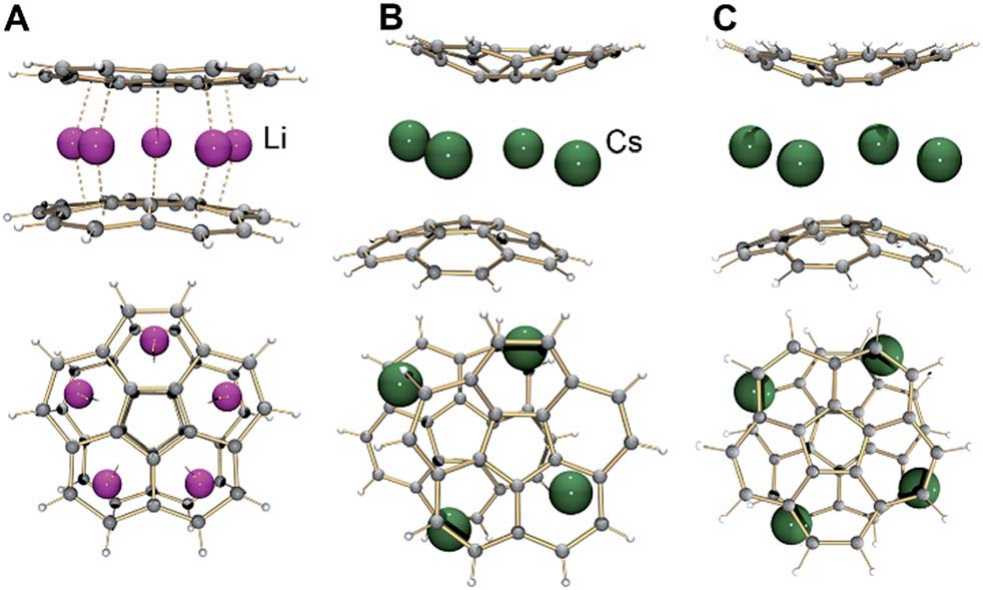
Side and top views of [(C_20_H_10_^4–^)/5Li^+^/(C_20_H_10_^4–^)]^3–^ (A) and [(C_20_H_10_^3–^)/4Cs^+^/(C_20_H_10_^3–^)]^2–^ (B) based on their X-ray diffraction studies along with the optimized geometry of the Cs_4_-sandwich based on DFT calculations (C, exterior Cs ions are not shown).

It has been previously proven that the highly reduced corannulene tetraanions form the triple-decker supramolecular aggregates with lithium counterions, [(C_20_H_10_^4–^)/5Li^+^/(C_20_H_10_^4–^)]^3–^.21a,b In the latter, five small lithium ions are encapsulated between the convex faces of two corannulene anions (Fig. 2A). Based on earlier NMR investigations,^28^ it was speculated that such aggregation is not favored for C_20_H_10_^4–^ anions in the presence of the heavier congeners of lithium.

In contrast to tetrareduced corannulene, NMR spectroscopy could not be used for predicting the self-aggregation pattern of the trianion-radical, leaving single crystal X-ray diffraction as the only source of structural information in this case.

In **1**, a convex-to-convex arrangement of two bowls is found with their shortest separation of 4.952(5) Å. However, in contrast to an almost ideally eclipsed bowl overlap in [(C_20_H_10_^4–^)/5Li^+^/(C_20_H_10_^4–^)]^3–^, the [(C_20_H_10_^3–^)/4Cs^+^/(C_20_H_10_^3–^)]^2–^ aggregate exhibits a staggered conformation of two C_20_H_10_˙^3–^ anions, which are slipped in respect to each other by 1.843(5) Å (Fig. 2B). The sandwiched Cs2, Cs2′, Cs3, and Cs3′ ions form a rectangle with the Cs···Cs separations of 4.181(4) and 4.995(4) Å. The Cs2 ions are bound to both anionic bowls with the Cs2···C interatomic distances of 3.275(5)–3.740(5) Å (Cs2···C_6(centroid)_ 3.229(5) and 3.265(5) Å). The Cs3 ions exhibit noticeably shorter Cs3···C distances of 3.231(5)–3.775(5) Å (Cs3···C_6(centroid)_ is 3.188(5) Å). The binding of the sandwiched alkali metal ions and corannulene trianions can be best described as the electrostatic gluing of the Cs_4_^4+^-unit between two highly-charged polyaromatic surfaces, as shown below by theoretical calculations. The coordination spheres of the encapsulated Cs2 and Cs3 ions are completed by the chelating diglyme molecules (Fig. 1C). The corresponding Cs···O bond lengths (3.069(4)–3.466(4) Å) are close to those previously reported for the salts of aromatic ligands with cesium ions solvated by O-donors.20a,^29^

The extraneous Cs1 cation occupies the concave cavity of the anionic corannulene dish and is located above its five-membered ring with the Cs···C_hub_ distances measured at 3.116(5)–3.328(5) Å (Fig. 1B). The concave placement of metals into π-bowls is rare.^30^ For corannulene, the first *endo*-bound complex was reported in 2011 for the cesium salt of mono-anion, [Cs^+^(18-crown-6)(C_20_H_10_^–^)].20*a*

**Fig. 3:**
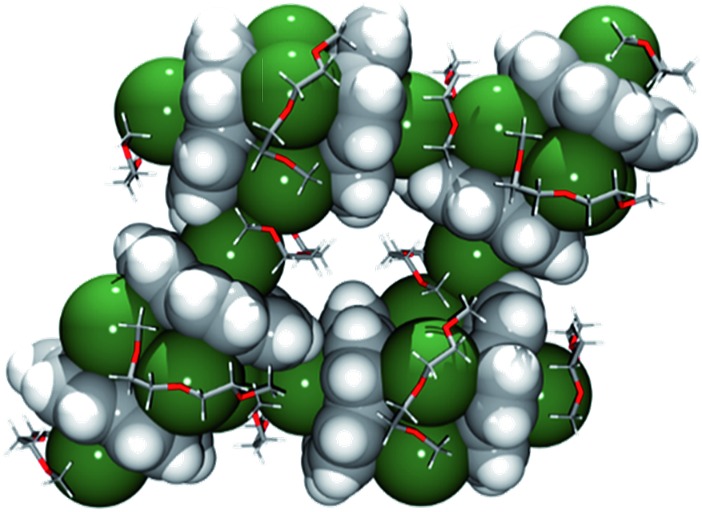
Fragment of a 2D polymeric network in **1**. Corannulene trianions and cesium cations are depicted using the space-filling model.

The preferential *endo*-binding of cesium ions to the corannulene bowl has been later observed in several other products.^26^,^31^ In **1**, the *endo* Cs···C_hub_ contacts with the triply-charged bowl are much shorter than those with C_20_H_10_˙^–^ in the above complex (3.424(3)–3.573(3) Å).20*a* In contrast to a very symmetrical cesium coordination in [Cs^+^(18-crown-6)(C_20_H_10_^–^)], the Cs1 ion in **1** is side-shifted toward the benzene rings of the corannulene trianion. The resulting intramolecular contacts between Cs1 and peripheral C-atoms of the bowl range from 3.275(5) to 3.662(5) Å. In addition, this Cs1 ion also shows binding (Cs···C 3.542(5)–3.638(5) Å) to the exterior of C_20_H_10_˙^3–^ from the neighboring unit and shares a diglyme molecule with the sandwiched Cs2 cation. The resulting Cs1···C and Cs1(2)···O interactions lead to the formation of a hybrid 2D polymeric network in the solid state of **1** (Fig. 3).

Interestingly, the acquisition of three electrons by C_20_H_10_ and aggregation with multiple cesium ions does not induce any significant flattening of the resulting trianion. The bowl depth of C_20_H_10_˙^3–^ in **1** (0.850(7) Å) remains almost unchanged compared to that in C_20_H_10_^0^ (0.875(2) Å).^32^ Although the addition of the first and second electrons to C_20_H_10_ is known to cause only minor core flattening (to 0.850(3) and 0.811(3) Å, respectively),^20^,^23^,^26^ a significant bowl depth reduction is observed upon four-electron acquisition (0.283(5)/0.329(5) Å,^21^Table 1). Thus, the carbon framework of C_20_H_10_˙^3–^, being more curved than those of the dianion and tetraanion of corannulene, stands out from the previously expected trend of a gradual flattening of the bowl upon consecutive electron addition.^33^ It is worth mentioning here that the theoretical value of the C_20_H_10_˙^3–^ bowl depth (0.41 Å) calculated earlier^34^ is twice smaller than the experimentally determined value found in **1**.

**Table 1:** Key distances [in Å] of corannulene and its anions, C_20_H_10_^*n*–^

*n* =	0^*a*^	1^*b*^	2^*c*^	3 (in **1**)	4^*d*^
	C_20_H_10_^0^	C_20_H_10_˙^–^	C_20_H_10_^2–^	C_20_H_10_˙^3–^	C_20_H_10_^4–^
Hub	1.411(2)–1.417(2)	1.389(5)–1.425(5)	1.390(3)–1.427(3)	1.401(7)–1.429(7)	1.391(5)–1.403(5)
Spoke	1.376(2)–1.381(2)	1.392(5)–1.417(5)	1.400(3)–1.418(3)	1.418(8)–1.443(7)	1.424(5)–1.432(5)
Flank	1.441(2)–1.450(2)	1.388(5)–1.462(5)	1.404(3)–1.475(3)	1.421(7)–1.439(7)	1.429(5)–1.443(5)
Rim	1.377(2)–1.387(2)	1.396(5)–1.453(6)	1.375(3)–1.435(3)	1.416(7)–1.438(8)	1.453(5)–1.462(5)
Bowl depth	0.875(2)	0.841(5)	0.811(3)	0.850(7)	0.283(5)/0.329(5)

^*a*^According to literature.^32^

^*b*^For [Li^+^(DME)_3_][C_20_H_10_^–^].^23^

^*c*^For [Li^+^(diglyme)_2_]_2_[C_20_H_10_^2–^].^23^

^*d*^For the naked [Li^+^_5_(C_20_H_10_^4–^)_2_]^3–^ sandwich.21*a*

The structural characterization of the previously missing corannulene trianion allowed us to compare for the first time the induced geometrical changes of C_20_H_10_˙^3–^ with those of C_20_H_10_^2–^ and C_20_H_10_^4–^ (Table 1). The central hub C–C bond lengths in trianion (1.401(7)–1.429(7) Å) are comparable to those measured in C_20_H_10_^2–^ (1.390(3)–1.427(3) Å)^20^,^26^ and slightly elongated than in C_20_H_10_^4–^ (1.391(5)–1.403(5) Å).^21^ The spoke C–C bond lengths in C_20_H_10_^*n*–^ (*n* = 2–4) species are almost equidistant. The rim C–C bonds of C_20_H_10_˙^3–^ (1.416(7)–1.438(8) Å) are notably shorter than in C_20_H_10_^4–^ (1.453(5)–1.462(5) Å). In general, all C–C bonds of C_20_H_10_˙^3–^ demonstrate some equalization of the bond lengths compared to corannulene, its mono- and di-anions.

## Computational studies

Due to extreme sensitivity of crystalline product **1** toward traces of water and/or oxygen, many important questions regarding its electronic structure and even its ground state remained unanswered. In order to provide insights into the geometry and electronic structure of the title product, computational modeling was performed at the PBE0/def2-TZVP+ECP(Cs)//cc-pVDZ(C,H,O) level of theory (see the ESI† for details).

### Geometry

The discrete trianionic species, C_20_H_10_˙^3–^, is expected to have a doublet ground state with one unpaired electron. In the sandwich-type aggregate (Fig. 2C), two such bowl-shaped radicals can exhibit ferromagnetic or antiferromagnetic coupling (so-called open-shell singlet or OS-singlet state), or alternatively show no coupling. First, the model system [Cs^+^//(C_20_H_10_^3–^)/4Cs^+^/(C_20_H_10_^3–^)//Cs^+^] was optimized in its triplet state. The direct comparison of this discrete calculated structure, [Cs^+^//(C_20_H_10_^3–^)/4Cs^+^/(C_20_H_10_^3–^)//Cs^+^], with the extended solid state structure of **1** immediately revealed a significantly smaller shift along the sandwich axis in the former (0.453 *vs.* 1.843(5) Å or, in terms of the distance between the bowl centroids: 4.89 Å *vs.* 5.28 Å, respectively). As a result, the encapsulated Cs_4_-unit exhibits an essentially square geometry in the calculated structure with the Cs···Cs contacts of 4.668 and 4.805 Å (*Δ* = 0.137 Å). For comparison, the corresponding Cs···Cs distances in the X-ray crystal structure differ by *ca.* 0.814 Å. The observed differences between the geometries of calculated and experimental sandwich structures illustrate the importance of additional intermolecular interactions existing in the 2D network in the solid state of **1**. This is the first time when the role of intermolecular interactions at the sandwich exterior is clearly observed, as in previous cases such effects were found negligible.^21^ The significant deviation of the theoretical model from the experimental X-ray crystal structure prompted us to consider four different models, namely (i) the simplest fully-optimized [Cs^+^//(C_20_H_10_^3–^)/4Cs^+^/(C_20_H_10_^3–^)//Cs^+^] model (**1**-*small*), (ii) the same model, but with the core structure taken from the X-ray experiment and kept unchanged, while positions of hydrogen atoms were optimized (**1H**-*small*), (iii) the fully-optimized model, in which all solvent molecules were considered explicitly (**1**-*full*), and (iv) the same system as in **1**-*full*, but with only hydrogen atom positions been optimized (**1H**-*full*), while the rest was taken from the crystal structure and kept frozen. Selecting the above four models (further details and all model structures are provided in the ESI†), we planned to evaluate the influence of coordinated solvent molecules and crystal packing on the ground state and electronic structure of the supramolecular sandwich-type aggregates formed by C_20_H_10_˙^3–^ with cesium ions.

Indeed, the fully relaxed geometry of a system with all coordinated solvent molecules (**1**-*full*) shows much closer resemblance with that of the X-ray crystal structure of **1**. For instance, the distance between the bowl centroids in **1**-*full* is equal to 5.38 Å and that is close to the experimental value of 5.28 Å. The difference in Cs···Cs contacts was found to be *ca*. 0.8 Å, which is essentially the same as in the experimental X-ray sandwich structure.

Notably, the calculated bowl depth values of corannulene trianion in the discrete model sandwich complexes with cesium ions are only slightly smaller than the experimental value (0.832 Å in **1**-*small* and 0.845 Å in **1**-*full vs.* 0.850(7) Å, respectively). This illustrates good approximation provided by the selected level of DFT calculations but also raises questions about the reasons for such unexpectedly high curvature of C_20_H_10_˙^3–^ in the [Cs^+^//(C_20_H_10_^3–^)/4Cs^+^/(C_20_H_10_^3–^)//Cs^+^] type of sandwiches. We have found that replacement of the concave bound Cs ions by much smaller Li ions to form the [Li^+^//(C_20_H_10_^3–^)/4Cs^+^/(C_20_H_10_^3–^)//Li^+^] sandwich results only in minor flattening of the bowl depth (0.775 Å), thus indicating that the effect of the outside cations is rather weak and the size of the sandwiched belt comprised of four large cesium ions may be the main reason for the observed curvature of corannulene trianion.

### Ground state

For the next step, we looked into the ground state of this unique sandwich-type assembly. Both abovementioned possible states, triplet and open-shell singlet, were examined. Broken-symmetry DFT (BS-PBE0 in our case) solution reveals small preference of the triplet state for the **1H**-*small* (*J* = +3.84 cm^–1^), **1**-*full* (+2.75 cm^–1^), and **1H**-*full* (+0.54 cm^–1^) models, whereas for **1**-*small* the OS-singlet state was found to be slightly lower in energy (–2.39 cm^–1^). However, the accuracy of these calculations does not allow an unambiguous conclusion about the actual ground state to be made. Therefore, we turned to the highly accurate multireference Møller–Plesset perturbation theory of the second order (MRMP2). The active space for the reference CASSCF wavefunction included six electrons over four orbitals (CASSCF(6,4) approach, see the ESI† for details). Calculations performed at the MRMP2 level revealed that the energy gap between the OS-singlet and triplet states is only –0.61 cm^–1^ for **1**-*small* and –0.02 cm^–1^ for **1H**-*small*. Subsequent extension of the active space to 14 electrons and 8 orbitals, CASSCF(14,8), resulted in a very similar outcome (+0.002 cm^–1^ and –0.01 cm^–1^, respectively). These findings revealed the absence of any significant magnetic coupling between the two C_20_H_10_˙^3–^ bowls within the triple-decker sandwich. It now could explain why the shift of two triply-reduced corannulene decks in respect to each other does not lead to changes in the nature of the ground state of the sandwich.

Notably, for this large and complex system the BS-DFT approach gives reasonably good results, correlating well with the more accurate MRPT2 method, and thus it can be considered as computationally “cheap” alternative to usually more accurate but significantly more demanding multireference approaches. Although the use of the multireference technique for full models (**1**-*full* and **1H**-*full*) is not feasible, we believe that the trend (no magnetic coupling between bowls as defined by BS-DFT method) is still valid and the main conclusion remains unchanged.

### Electronic structure

**Fig. 4:**
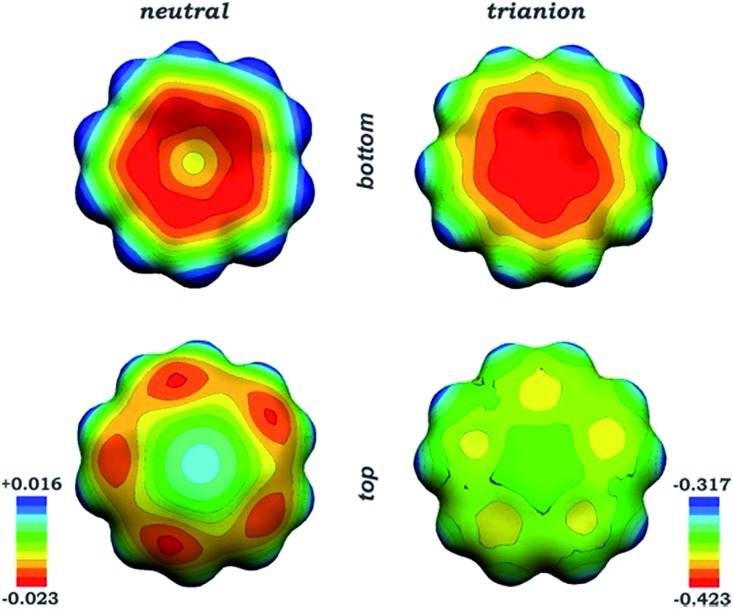
Molecular electrostatic potentials (MEPs) of the neutral C_20_H_10_ molecule (*left*) and discrete C_20_H_10_˙^3–^ anion (*right*).

Based on our previous findings, only triplet electronic configuration is considered hereafter for the sandwich aggregates. To begin with, the direct comparison of the electronic structure of neutral corannulene (singlet ground state) with the “naked” triply-reduced corannulene species was performed. In the discrete C_20_H_10_˙^3–^ anion, one unpaired electron is delocalized over the bowl core in accordance with topology of LUMO+1 of the neutral C_20_H_10_ bowl, thus making the curved polyaromatic surface to be uniformly more negatively charged (Fig. 4).

Comparison of charge distribution in the “naked” C_20_H_10_˙^3–^ bowl with that in the supramolecular sandwich revealed a notable redistribution of electron density or polarization of the corannulene trianions in the latter. Specifically, the interior part of the bowl (hub C-atoms) becomes more negatively charged, whereas the exterior part (rim C-atoms) appears to show negative charge depletion. For instance, the calculated atomic charges of the hub C-atoms range from –0.134 to –0.176 and from –0.144 to –0.171 for models **1**-*small* and **1H**-*small*, respectively, *vs.* –0.10 charges in the discrete C_20_H_10_˙^3–^ species. This effect can naturally be assigned to the presence of the positively charged belt of cesium cations jammed between two triply-reduced corannulene bowls. Accounting for coordinated solvent molecules resulted in a slightly less pronounced redistribution of the negative charge (–0.126 to –0.153 and –0.126 to –0.151 in **1**-*full* and **1H**-*full* models). These observations indicate that coordination of solvent molecules by metal cations, albeit influencing the geometry, does not significantly disturb the electronic structure of the supramolecular aggregate.

**Fig. 5:**
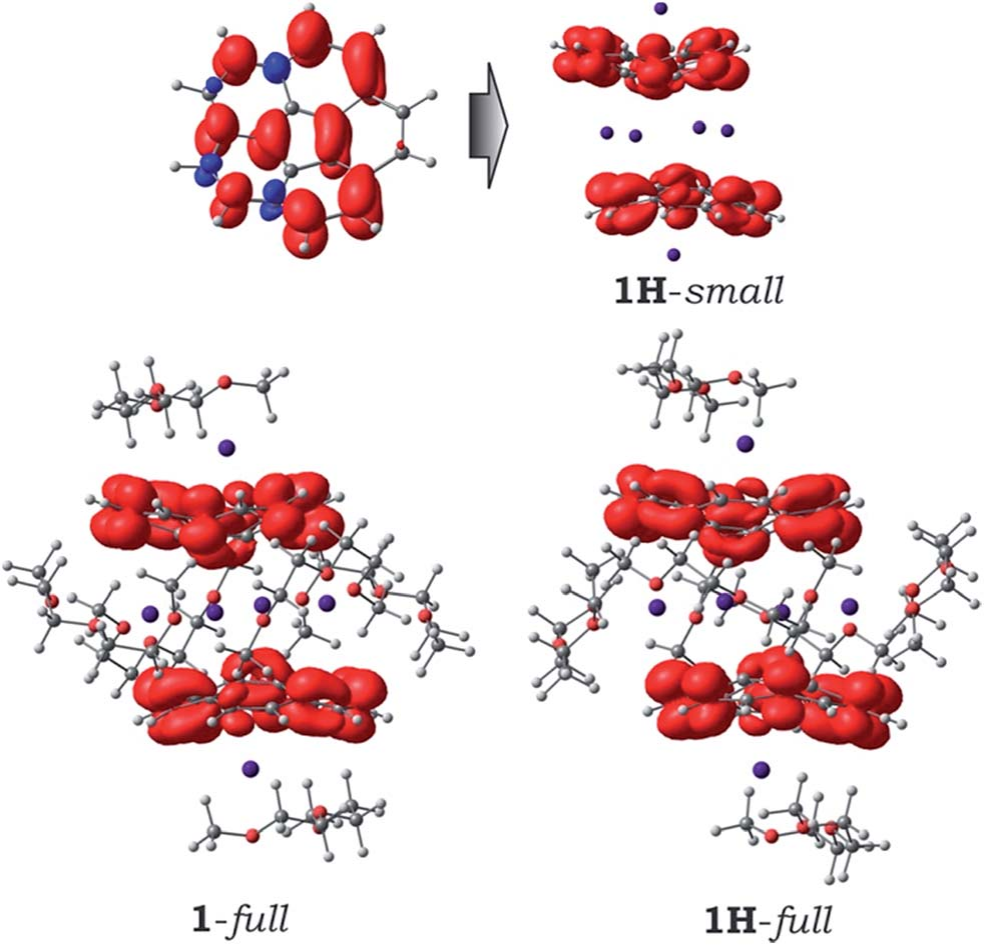
Spin density distribution (0.002 a.u. isosurface) for the discrete C_20_H_10_˙^3–^ anion (*top left*), **1H**-*small* model (*top right*), and **1**-*full* and **1H**-*full* models (*bottom*).

Despite the redistribution of atomic charges when going from the “naked” C_20_H_10_˙^3–^ species to [Cs^+^//(C_20_H_10_^3–^)/4Cs^+^/(C_20_H_10_^3–^)//Cs^+^] aggregate, the spin density in the latter remains essentially the same (Fig. 5), showing the presence of two uncoupled corannulene trianion-radicals in the system. Moreover, the topology of spin density is not notably influenced by the presence of solvent molecules and/or by crystal packing effects (as shown by comparison of models **1H**-*small*, **1**-*full* and **1H**-*full*, Fig. 5). This finding is in complete agreement with the previous conclusion about ground state of such supramolecular aggregates.

### Supramolecular assemblies formed by corannulene trianion *vs.* tetraanion

This work revealed for the first time an ability of C_20_H_10_˙^3–^ to form sandwich-type supramolecular assembly with alkali metals, allowing us to provide the direct comparison of electronic structures and bonding in the remarkable organometallic aggregates formed by trianion *vs.* tetraanion of corannulene. The highly reduced C_20_H_10_^4–^ anion was previously shown to afford triple-decker supramolecular assemblies with encapsulated belts of alkali metal cations, several of which were crystallographically characterized and their core electronic structures were probed by theoretical methods.21a,b Notably, the C_20_H_10_^4–^ anions in such sandwich products were found to be strongly coupled with a singlet ground state, showing significant delocalization of corresponding molecular orbitals (MOs) between the two polyaromatic bowls. Moreover, it was found that such coupling is responsible for the record ^7^Li-NMR negative chemical shift observed for the internally encapsulated lithium cation in the heterobimetallic alkali metal sandwiches.^22^ In contrast, the aggregates formed by C_20_H_10_˙^3–^ show no coupling between the corannulene moieties, as confirmed by the high-level theoretical modeling in this study. In this regard, a direct comparison of these two types of supramolecular sandwich-type assemblies should be interesting and informative.

Careful checking of MOs in [Cs^+^//(C_20_H_10_^3–^)/4Cs^+^/(C_20_H_10_^3–^)//Cs^+^] (all models) indeed revealed the absence of any orbital responsible for the coupling between the two triply-reduced corannulene bowls, in contrast to the coupling previously found in the triple-decker sandwiches formed by the tetraanions.

**Fig. 6:**
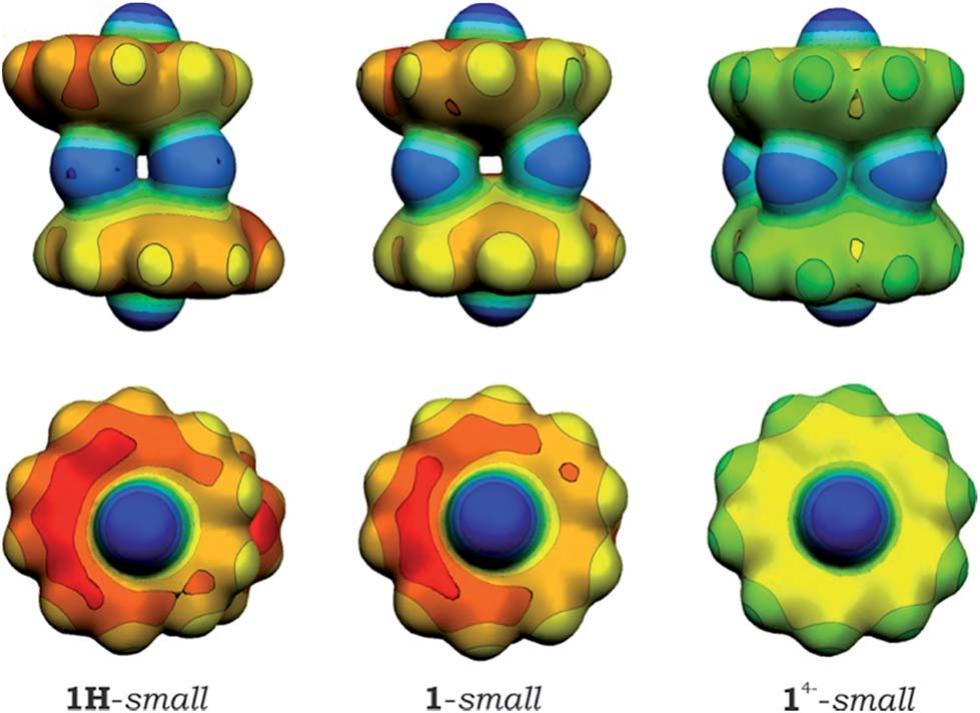
Molecular electrostatic potentials (MEPs) for the **1H**-*small* (*left*), **1**-*small* (*center*), and **1**^4–^-*small* (*right*) models.

Subsequent analysis of charge distribution in **1H**-*small* and **1**-*small* models and their comparison with a sandwich formed by C_20_H_10_^4–^ anions, [Cs^+^//(C_20_H_10_^4–^)/5Cs^+^/(C_20_H_10_^4–^)//Cs^+^]^–^ (**1**^4–^-*small*), revealed that the interior part of the latter aggregate is significantly more negatively charged with a very narrow range of atomic charges ranging from –0.179 to –0.180 for hub C-atoms. Similar equalization of atomic charges was also observed for rim C-atoms (from –0.387 to –0.388) as well as for spoke ones (from –0.182 to –0.183), which can be attributed to the electron density sharing due to coupling between two tetrareduced corannulene bowls. In the [Cs^+^//(C_20_H_10_^3–^)/4Cs^+^/(C_20_H_10_^3–^)//Cs^+^] systems, such equalization was not found and the range of atomic charges for carbon atoms of the same type is notably wider. This observation is illustrated graphically in Fig. 6, which unambiguously shows that non-compensated negative charge is still localized on the bowl-shaped fragments in [Cs^+^//(C_20_H_10_^3–^)/4Cs^+^/(C_20_H_10_^3–^)//Cs^+^] aggregates. Interestingly, both models, **1**-*small* and **1H**-*small*, show the same features in MEP maps, again confirming the absence of notable coupling between the C_20_H_10_˙^3–^ bowls.

At the same time, the NBO charge distribution analysis revealed the positive charge of cesium centers to be very close to 1 (average value for four sandwiched cations is +0.92 and for two exterior ions is +0.96) in all considered Cs_4_-sandwich models. Interestingly, the charges of interior cesium ions in the Cs_5_-sandwich (**1**^4–^-*small* model, calculated at the same level of theory) were also found to be +0.93. The total charge of the computed Cs_5_-sandwich of –3 also shows essentially no charge transfer or charge delocalization to/from the encapsulated cesium ions. Thus, the positively charged belt of alkali metal cations plays the role of “electrostatic glue” in both types of sandwich aggregates. The main difference between the trianion- and tetraanion-based supramolecular products is the coupling of two negatively charged corannulene decks, which is not observed in the former and found to be significant in the latter.

In order to evaluate the coupling and thus to provide further insights into the electronic structure and bonding in these unusual supramolecular systems, the Energy Decomposition Analysis (EDA) has been performed. The results are summarized in Table 2.

**Table 2:** Results of EDA analysis for **1**-*small*, **1**-*full* and **1**^4–^-*small* models ((PBE0/TZ2P/ZORA) in kcal mol^–1^)

Parameters	**1**-*small*	**1**-*full*	**1** ^4–^-*small*
DE_int_	–942.57	–786.63	–1536.03
DE_elstat_	–853.45	–760.52	–1452.97
DE_orb_	–241.89	–200.84	–341.41
DE_Pauli_	+152.77	+174.73	+258.35

In the EDA analysis we used the following fragmentation scheme. For **1**-*small*, three interacting fragments were considered, namely two [(Cs)(C_20_H_10_)]^2–^ and one [Cs_4_]^4+^ species. For **1**-*full* model, all metal cations (including the external Cs^+^ ions as well as ions jammed between two bowls) were coordinated by solvent molecules, similar to the X-ray crystal structure. In the case of **1**^4–^-*small*, these three fragments included two [(Cs)(C_20_H_10_)]^3–^ and one [Cs_5_]^5+^ species. Such fragmentation allows one to tentatively evaluate interactions in the supramolecular aggregates as well as to get estimates of coupling between the negatively charged bowls. As shown in Table 2, the electrostatic contribution is dramatically greater in **1**^4–^-*small* system in comparison with that in **1**-*small* and/or **1**-*full* ones. This result should be expected due to the difference in charges of bowl-shaped fragments (4- *vs.* 3-) and the number of Cs^+^ ions sandwiched in-between. At the same time, the orbital term, which is usually interpreted as a covalent contribution, is also greater by *ca*. 100 kcal mol^–1^ in **1**^4–^-*small* system. This large difference can be naturally attributed to the presence of a notable coupling between the two polyaromatic bowls in the latter. Altogether, these contributions (DE_elstat_ and DE_orb_) make supramolecular systems based on tetrareduced corannulene significantly more stable than those based on C_20_H_10_˙^3–^ anions. Note, this general trend is not altered even with some increase in Pauli repulsion in the same direction (DE_Pauli_ term in Table 2). This conclusion is in perfect agreement with experimental findings of this work as well as with previous studies.

## Conclusions

The triply-reduced corannulene, that was previously missing in the family of consecutively generated negatively charged C_20_H_10_^*n*–^ anions (*n* = 1–4), has now been isolated as the crystalline cesium salt, [Cs^+^_3_(diglyme)_2_(C_20_H_10_^3–^)]. The X-ray crystallographic characterization of the product demonstrated that corannulene trianions form a novel supramolecular aggregate with four large cesium ions sandwiched between two bowls in [(C_20_H_10_^3–^)/4Cs^+^/(C_20_H_10_^3–^)]^2–^. The double negative charge of the resulting triple-decker sandwich is compensated by two extraneous Cs^+^ cations that fill the concave cavities of both corannulene bowls. This supramolecular trapping of triply-reduced corannulene in the “cesium-sealed” self-assembly seems required in order to catch this transient species, as multiple previous attempts to isolate C_20_H_10_˙^3–^ with small lithium ions have been unsuccessful so far.

This first crystallographic characterization of the C_20_H_10_˙^3–^ anion allowed us to follow the effect of adding three electrons to a π-bowl and to provide structural comparison for the whole series of successively generated C_20_H_10_^*n*–^ anions.

The isolation of the crystalline product **1** also provided the first reliable UV-vis spectroscopic characterization of C_20_H_10_˙^3–^ based on comparison of the spectra for dissolved crystals with the *in situ* generated trianion. These data can now be used for the detection of the triply-reduced state of corannulene in solutions. Finally, these new results expand the unique organometallic and supramolecular chemistry of the highly charged bowl-shaped polyaromatic carbanions.^21^,^22^,^35^ For the first time, we show that the triply-reduced corannulene also has an ability to stabilize a high-nuclearity alkali metal layer, as previously observed for the very electron-rich corannulene tetraanions.

## Supplementary Material

Supplementary information

Crystal structure data
