# Supplementary material for: Supramolecular trap for a transient corannulene trianion
Source: Chem Sci. 2015 Dec 17;7(3):1954–61. doi: 10.1039/c5sc04385a (PMC5966907; doi:10.1039/c5sc04385a)
Supplement: Supplementary file 1 [file SC-007-C5SC04385A-s001.pdf]

Electronic Supplementary Material (ESI) for Chemical Science.
This journal is © The Royal Society of Chemistry 2015

Supporting Information

Supramolecular Trap for a Transient Corannulene Trianion

Alexander V. Zabula,[a,b] Sarah N. Spisak,[a] Alexander S. Filatov,[a] Andrey Yu. Rogachev,[c,*]

Rodolphe Clérac,[d,e] Marina A. Petrukhina[a,*]

[a] Department of Chemistry, University at Albany, State University of New York, Albany, NY 12222-0100 (USA)
[b] Department of Chemistry, University of Pennsylvania, Philadelphia, PA 19104 (USA)
[c] Department of Chemistry, Illinois Institute of Technology, Chicago, IL 60616 (USA)
[d] CNRS, CRPP, UPR 8641, F-33600 Pessac (France)
[e] Univ. Bordeaux, CRPP, UPR 8641, F-33600 Pessac (France)

* mpetrukhina@albany.edu; arogache@iit.edu

Materials and Methods S3
Crystallization of [Cs^+_3(diglyme)$_2$($C_{20}H_{10}^{3-}$)] (1) S4
Magnetic Measurements S4
ESR Data S4
Figure S1. Experimental ESR spectrum of $C_{20}H_{10}^{\bullet 3-}$ generated by cesium reduction S5
Crystal Structure Determinations and Refinement of 1 S5
Table S1. Crystallographic data for **1** S5
Figure S2. UV-vis spectrum of **1** (in diglyme) S6
Figure S3. UV-vis spectra of *in-situ* generated products in diglyme using (a) excess Cs and (b) excess Li and Cs (3.5 eq.) S7
Theoretical Calculations S7
Figure S4. Equilibrium structures for models **1**-*small*, **1H**-*small*, and **1**$^{4-}$-*small* S8
Figure S5. Equilibrium structures for models **1**-*full* and **1H**-*full* S8
Figure S6. Equilibrium structures for (a) "naked" trianion $C_{20}H_{10}^{\bullet 3-}$, and (b) neutral corannulene (PBE0/cc-pVDZ) S9
Figure S7. CASSCF(6,4) natural orbitals along with occupancies for **1**-*small* model S10
Figure S8. CASSCF(14,8) natural orbitals along with occupancies for **1**-*small* model S11
Figure S9. CASSCF(6,4) natural orbitals along with occupancies for **1H**-*small* model S11
Figure S10. CASSCF(14,8) natural orbitals along with occupancies for **1H**-*small* model S12
Table S2. Absolute energies of **1**-*small*, **1H**-*small*, **1**-*full*, and **1H**-*full* as well as of "naked" trianion $C_{20}H_{10}^{3-}$ (PBE0/def2-TZVP(Cs)//cc-pVDZ(C,H,O)) S12
Table S3. Absolute energies of all systems as results of broken-symmetry calculations (PBE0/TZVP/ZORA) S12
Table S4. Absolute energies of **1**-*small* and **1H**-*small* systems as results of MRMP2 calculations based on different converged CASSCF wavefunctions (CAS(6,4) and CAS(14,8)) S13
Figure S11. LUMO+1 for neutral $C_{20}H_{10}$ molecule (PBE0/cc-pVDZ) S13
Table S5. Cartesian coordinates for **1**-*small* system, optimized at the PBE0/def2-TZVP(Cs)//cc-pVDZ(C,H,O) level of theory S14

Table S6. Cartesian coordinates for **1**$^{4–}$-*small* system, optimized at the PBE0/def2-TZVP(Cs)//cc-pVDZ(C,H,O) level of theory S15
Table S7. Cartesian coordinates for **1H**-*small* system, optimized at the PBE0/def2-TZVP(Cs)//cc-pVDZ(C,H,O) level of theory S16
Table S8. Cartesian coordinates for **1**-*full* system, optimized at the PBE0/def2-TZVP(Cs)//cc-pVDZ(C,H,O) level of theory S17
Table S9. Cartesian coordinates for **1H**-*full* system, optimized at the PBE0/def2-TZVP(Cs)//cc-pVDZ(C,H,O) level of theory S19
Table S10. Cartesian coordinates for “naked” trianion $C_{20}H_{10}^{•3–}$, optimized at the PBE0/cc-pVDZ level of theory S22
Table S11. Cartesian coordinates for neutral $C_{20}H_{10}$, optimized at the PBE0/cc-pVDZ level of theory S23
Table S12. NBO charges for **1**-*small* model (PBE0/def2-TZVP(Cs)//cc-pvDZ(C,H,O)) S23
Table S13. NBO charges for **1**-*small* model (PBE0/def2-TZVP(Cs)//cc-pvDZ(C,H,O)) S24
Table S14. NBO charges for **1**$^{4–}$-*small* model (PBE0/def2-TZVP(Cs)//cc-pvDZ(C,H,O)) S25
Table S15. NBO charges for **1**-*full* model (PBE0/def2-TZVP(Cs)//cc-pvDZ(C,H,O)) S26
Table S16. NBO charges for **1H**-*full* model (PBE0/def2-TZVP(Cs)//cc-pvDZ(C,H,O)) S29
Table S17. NBO charges for “naked” $C_{20}H_{10}^{•3–}$ (PBE0/cc-pvDZ) S32
Table S18. NBO charges for neutral $C_{20}H_{10}$ (PBE0/cc-pvDZ) S32
EDA Analysis of Sandwich-Type Aggregates S33
Figure S12. EDA Fragmentation scheme in **1**-*small* and **1H**-*small* models (*left*) as well as for **1**$^{4–}$-*small* (*right*) S33
Figure S13. EDA Fragmentation scheme in **1**-*full* and **1H**-*full* models S34
References S34

Materials and Methods.

All manipulations were carried out using break-and-seal[1] and glove-box techniques under an atmosphere of argon. THF and hexanes were dried over Na/benzophenone and distilled prior to use. Diglyme was dried over NaK_2 alloy and vacuum-transferred. Lithium and dicyclohexano-18-crown-6 (99%) were purchased from Sigma-Aldrich. Cesium was purchased from Strem Chemicals. Crown ether was dried over P_2O_5 *in vacuo* for 24 hours. Corannulene was prepared as described previously[2-4] and sublimed at 175 °C prior to use. The UV-vis spectra were recorded on a PerkinElmer Lambda 35 spectrometer. The ESR spectra were recorded on a Bruker ER-200 D-SRC X-band spectrometer that is interfaced to a Compaq 386 PC equipped with the IBM analog-to-digital converter and Scientific Software Services Systems (Bloomington, IL). The trianion of corannulene was generated by the reaction of cesium metal or Li/Cs mixture (excess) with the solution of $C_{20}H_{10}$ in THF ($\approx 4\times10^{-4}$ M) in the sealed quartz capillary (∅ 1.0 mm) at 25 ºC. The probe for the ESR study was also prepared by dissolving crystals of **1** and transferring the resulting solution ($\approx 2\times10^{-5}$ M) into the quartz capillary (∅ 0.6 mm), which was then sealed. Magnetic measurements were performed on polycrystalline samples of **1** (between 10 and 20 mg). The samples were manipulated in a drybox under nitrogen atmosphere and sealed in a polyethylene bag (3 × 0.5 × 0.02 cm; between 20 and 35 mg) in order to collect data in the temperature range of 1.8 to 300 K at 1000 Oe. Magnetic measurements were obtained with the use of a Quantum Design SQUID magnetometer MPMS-XL functioning between 1.8 and 400 K for direct-current (dc) applied fields ranging from -7 to +7 T. Prior to the experiment, the field-dependent magnetization was measured at 100 K on the sample in order to prove the absence of any bulk ferromagnetic impurities. The magnetic data were corrected for the sample holder and diamagnetic contributions. Elemental analysis was performed by Complete Analysis Laboratories, Inc., Parsippany, NJ.

Crystallization of $[Cs^{+}{}_{3}(diglyme)_{2}(C_{20}H_{10}{}^{3-})]$ (1).

Direct Cs reduction. Diglyme (3 mL) was added to a flask containing Cs metal (28 mg, 0.21 mmol, 3.5 eq.), corannulene (15 mg, 0.06 mmol), and dicyclohexano-18-crown-6 (22 mg, 0.06 mmol). The resulting green mixture was stirred at room temperature for 60 hours to give a bright purple-red solution. This solution was filtered, layered with hexanes (3 mL), and kept at 10 °C. A few X-ray quality crystals of **1** were collected in 4 weeks. Yield: 5.5 mg, 10%.

Cs/Li reduction. Diglyme (3 mL) was added to a flask containing Cs metal (28 mg, 0.21 mmol, 3.5 eq.), Li (0.85 mg, 0.12 mmol), and corannulene (15 mg, 0.06 mmol). The resulting green solution was stirred at room temperature for 16 hours to give a bright purple-red solution. This solution was filtered, layered with hexanes (3 mL), and kept at 10 °C. The X-ray quality crystals (prisms) of **1** were deposited in 72 hours. Crystals of **1** were collected, washed with hexanes (2 x 3 mL), dried and used for characterization. Yield: 19.3 mg, 35%.
UV-vis (diglyme, nm): λ_{max} = 388. UV-vis (THF, nm): λ_{max} = 386. Anal. Calcd for $C_{32}H_{38}Cs_{3}O_{6}$: C, 41.90; H, 4.18; Found: C, 41.78; H, 4.14.

Magnetic Measurements. Multiple samples of single crystals collected from the above reactions have been sent for magnetic measurements over the period of two years. All our attempts to collect good sets of magnetic data have failed likely due to extreme air- and moisture sensitivity of the title product. Our rough estimates point out to very weak interactions of corannulene trianions within the sandwich-type assemblies with an exchange coupling between -5 and -10 K, which makes a gap between the singlet ground state and the triplet state of about 10–20 K. This is consistent with our theoretical calculations showing degeneracy of the two states. Unfortunately, the absolute values of the susceptibility could not be obtained reliably.

ESR Data. Using probes prepared by dissolving the crystals of **1** in THF or diglyme, we were unable to detect any well-resolved ESR signal. Only in the presence of an excess of cesuim metal upon the *in situ* reduction of $C_{20}H_{10}$ in THF the highly resolved ESR signal centered at g = 2.0038 could be observed in 24 h (Figure S1). These ESR data point out to the formation of trianion-radicals in solution, consistent with previous works of Scott and coworkers.[5] However, it has to be mentioned here that we could not isolate the sandwich type aggregates from the THF solutions, as the title product is formed only when diglyme is used as a solvent media.

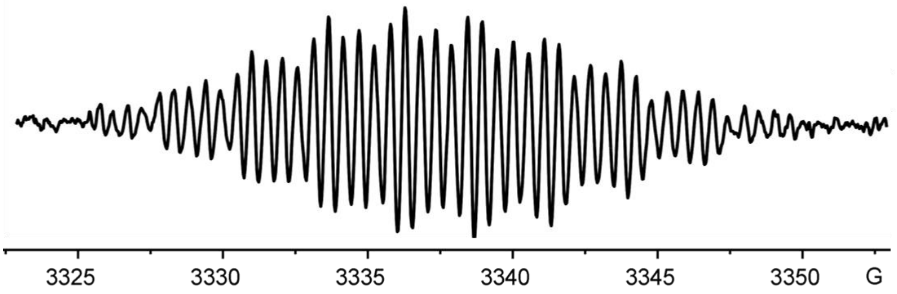

Figure S1. Experimental ESR spectrum of $C_{20}H_{10}^{\bullet 3-}$ generated by *in situ* cesium reduction.

Crystal Structure Determinations and Refinement of 1. Data collection was performed on a Bruker SMART APEX CCD-based X-ray diffractometer with graphite-monochromated Mo-Kα radiation (λ = 0.71073 Å) at T = 100(2) K. Data were corrected for absorption effects using the empirical method SADABS.[6] The structure was solved by direct methods and refined using the Bruker SHELXTL (Version 6.14) software package.[7] Hydrogen atoms were included at idealized positions using the riding model. For further crystal and data collection details see Table S1.

Table S1. Crystallographic data for **1**.

Parameter	**1**
Empirical formula	$C_{32}H_{38}Cs_3O_6$
M_r	917.35

Crystal system	monoclinic
Space group	*C*2/*c*
a (Å)	23.597(3)
b (Å)	14.073(2)
c (Å)	19.874(3)
α (º)	90
β (º)	93.283(2)
γ (º)	90
V (Å^3)	6589.1(16)
Z	8
ρ_{calcd} [g·cm^{-3}]	1.849
μ [mm^{-1}]	3.342
Reflections collected	27870
2θ-range [deg]	3.38−56.54
independent reflections, R_{int}	7687, 0.0465
data/restraints/parameters	7687/0/374
R_1,[a] wR_2[b] [I>2σ(*I*)]	0.0449, 0.0978
R_1,[a] wR_2[b] [all data]	0.0598, 0.1052
quality of fit[c]	1.090
peak/hole [e·Å^{-3}]	1.834/−1.062

[a] $R_1 = \Sigma||F_o|-|F_c||/\Sigma|F_o|$. [b] $wR_2 = [\Sigma[w(F_o^2-F_c^2)^2]/\Sigma[w(F_o^2)^2]]^{½}$. [c] Quality-of-fit = $[\Sigma[w(F_o^2-F_c^2)^2]/(N_{obs}-N_{params})]^{½}$, based on all data.

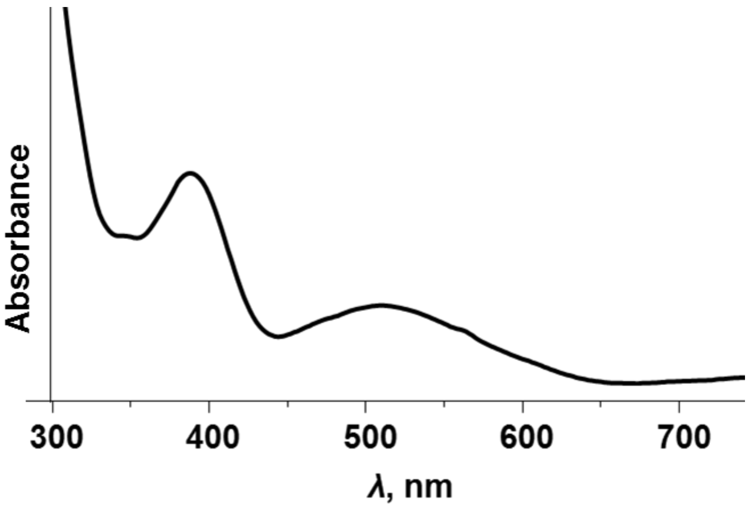

Figure S2. UV-vis spectrum of **1** (in diglyme).

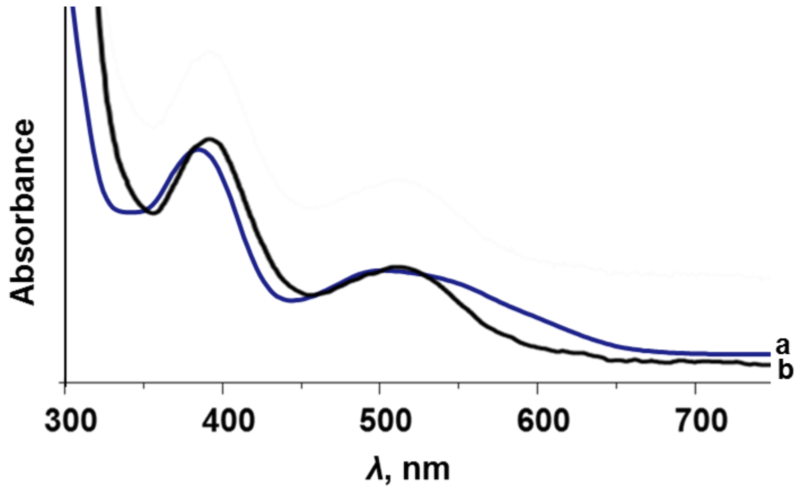

Figure S3. UV-vis spectra of *in-situ* generated products in diglyme using (a) excess Cs and (b) excess Li and Cs (3.5 eq.).

Theoretical Calculations

Geometry optimizations for systems **1**-*small*, $\mathbf{1}^{4-}$-*small*, and **1**-*full* (Figures S3-S4) were performed at the PBE0/def2-TZVP(Cs)//cc-pVDZ(C,H,O) level of theory. Geometry optimizations of the structures of neutral $C_{20}H_{10}$ and "naked" trianion $C_{20}H_{10}^{\bullet 3-}$ species (Figure S6) were carried out at the PBE0/cc-pVDZ level of theory. All calculations were carried out with the Firefly program package (version 8.1.0).[8] The calculated structures correspond to the local minimum (no imaginary frequencies) on the corresponding potential energy surfaces, as determined by calculation of the full Hessian matrix followed by estimation of frequencies in the harmonic approximation. In the case of **1H**-*small* and **1H**-*full* models, only positions of hydrogen atoms were optimized, whereas positions of other atoms were taken from the crystal structure and kept frozen (Figures S4-S5). These calculations were performed with the ORCA program suite (version 3.0.3).[9] In this part, all atoms were described by relativistically re-contracted basis sets of triple-ζ quality (SARS-TZVP).[10] All calculations were carried out with RIJCOSX acceleration technique.[11] Scalar relativistic effects have been incorporated by applying the 0th-order regular approximation (ZORA). All optimized geometries were then used for subsequent analysis of the electronic structure of the

product in terms of natural bond orbitals (NBO) approach.[12] All NBO computations were performed with the NBO 6.0 program.[13] Broken-symmetry (BS-PBE0) calculations were performed with help of ORCA package using Yamaguchi formula[14] for calculating J coupling constant.

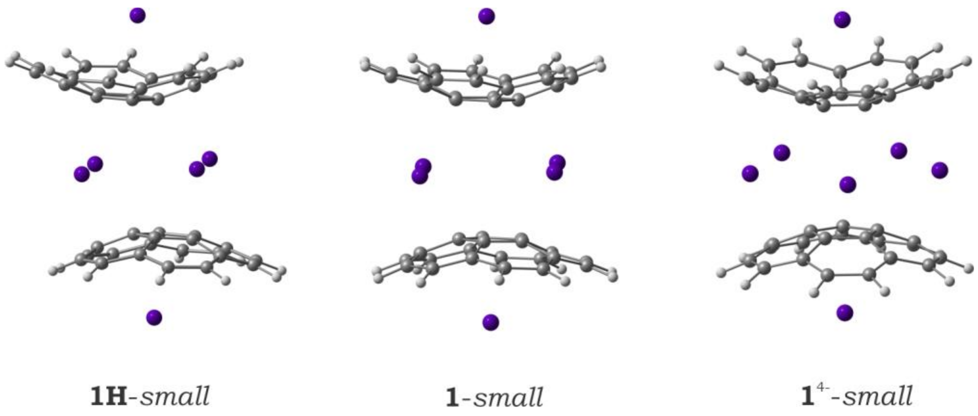

Figure S4. Equilibrium structures for models **1**-*small*, **1H**-*small*, and **1**$^{4-}$-*small*.

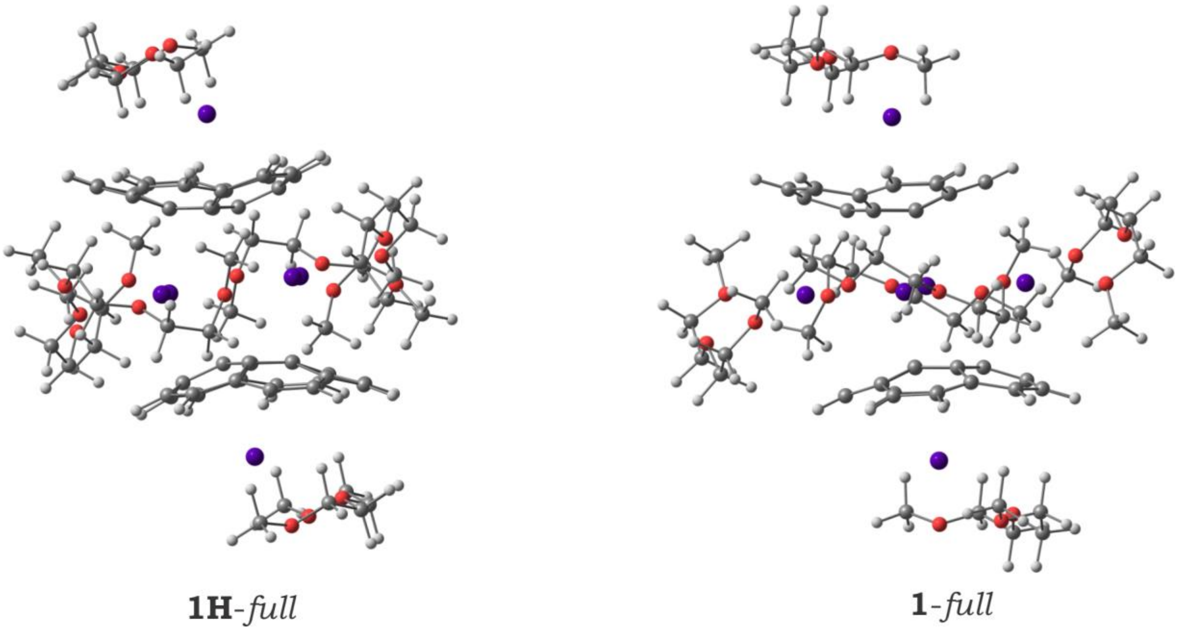

Figure S5. Equilibrium structures for models **1**-*full* and **1H**-*full*.

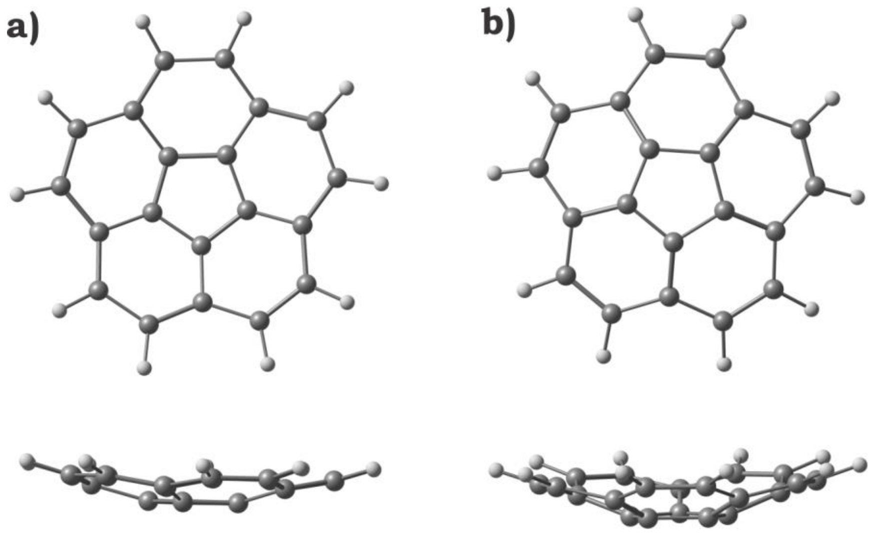

Figure S6. Equilibrium structures for (a) "naked" trianion $C_{20}H_{10}^{\bullet 3-}$, and (b) neutral corannulene (PBE0/cc-pVDZ).

Multireference calculations were performed at the level of multiconfigurational perturbation theory of the second in XMCQDPT2 variant.[15] The same basis sets were utilized as for the geometry optimization (def2-TZVP(Cs)//cc-pVDZ(C,H,O)). Two different active spaces, namely (6,4) and (14,8) were considered for reference CASSCF calculations. The first active space includes all doubly and singly occupied orbitals on the top of neutral corannulene and 6 cesium cations. This active space comes from the electronic structure of corannulene, which has two degenerate LUMOs of π-symmetry. These orbitals are populated when the system is reduced. Totally, this active space describes 6 electrons shared over 4 orbitals. The second active space (14 shared over 8 orbitals or 14/8 approach) was constructed by adding four occupied MOs, which represent two degenerate HOMOs of the neutral corannulene. These additional orbitals correspond to the bonding partners of LUMOs in $C_{20}H_{10}$. Both active spaces used in this study are depicted in Figures S7-S8 and Figures S9-S10 for systems **1**-*small* and **1H**-*small*, respectively. An initial guess of the orbitals for CASSCF calculations were taken from the converged PBE0 calculations. CASSCF calculations were performed using a state-average approach. The lowest-lying one singlet and

one triplet states were considered (with ALDET keyword in Firefly terminology). The singlet state was found corresponding to an open-shell singlet electronic state. The converged CASSCF wavefunction was further used as a reference wavefunction for the calculations by multireference Møller-Plesset perturbation theory of the second order (MRMP2). The MRMP2 model is a special state-specific case of the XMCQDPT2 theory that was recently developed by Granovsky.[15] The conventional intruder state avoidance (ISA) technique[16] was used in MRPT2 calculations.

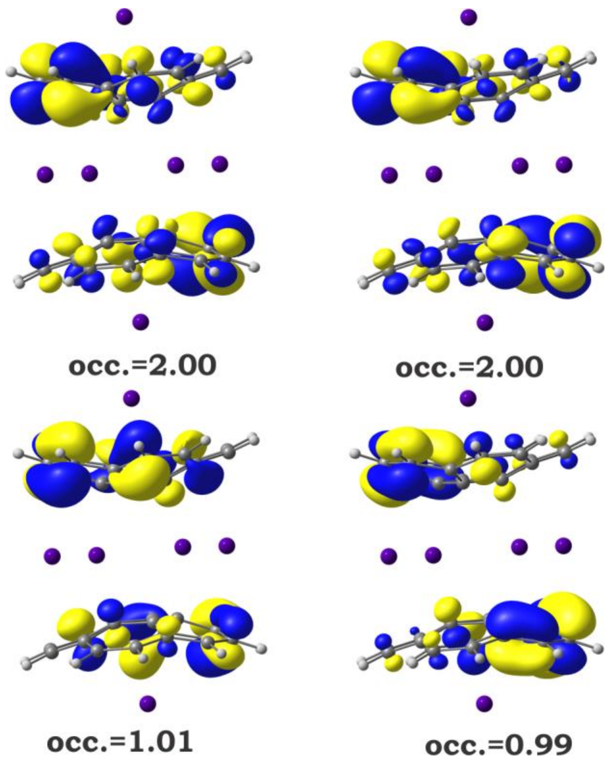

Figure S7. CASSCF(6,4) natural orbitals along with occupancies for **1**-*small* model.

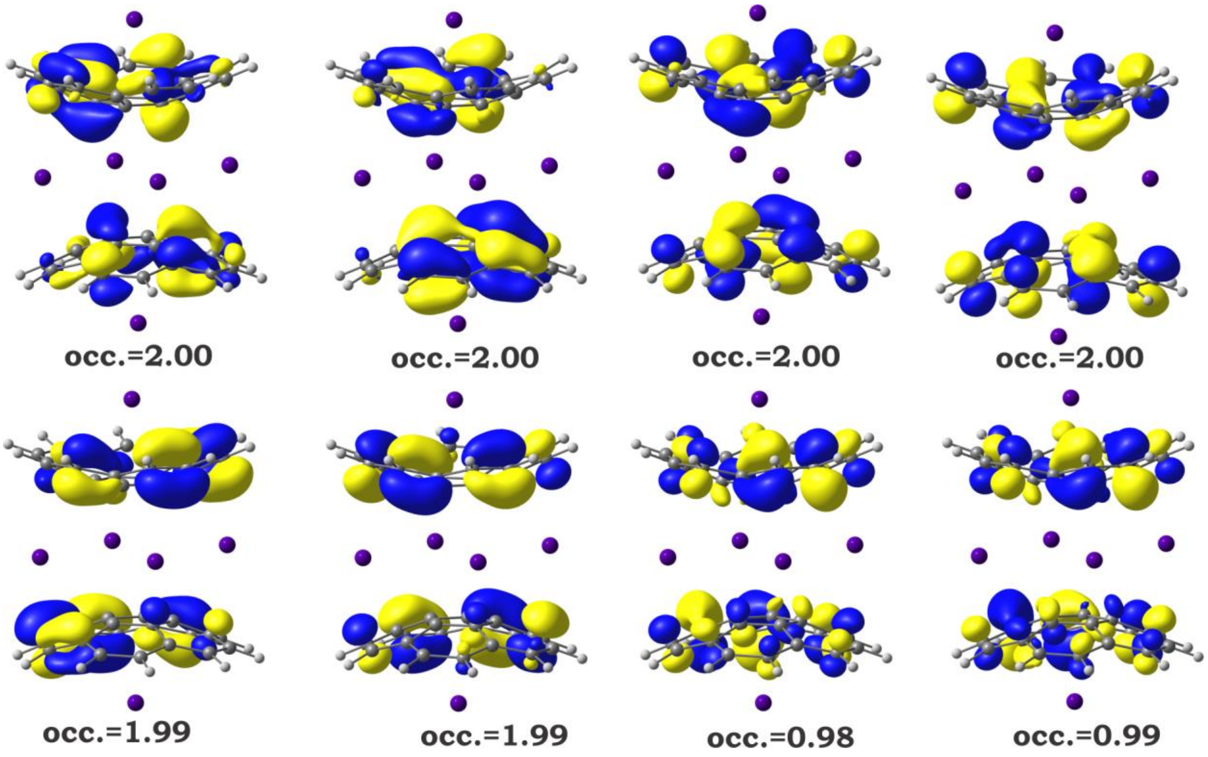

Figure S8. CASSCF(14,8) natural orbitals along with occupancies for **1**-*small* model.

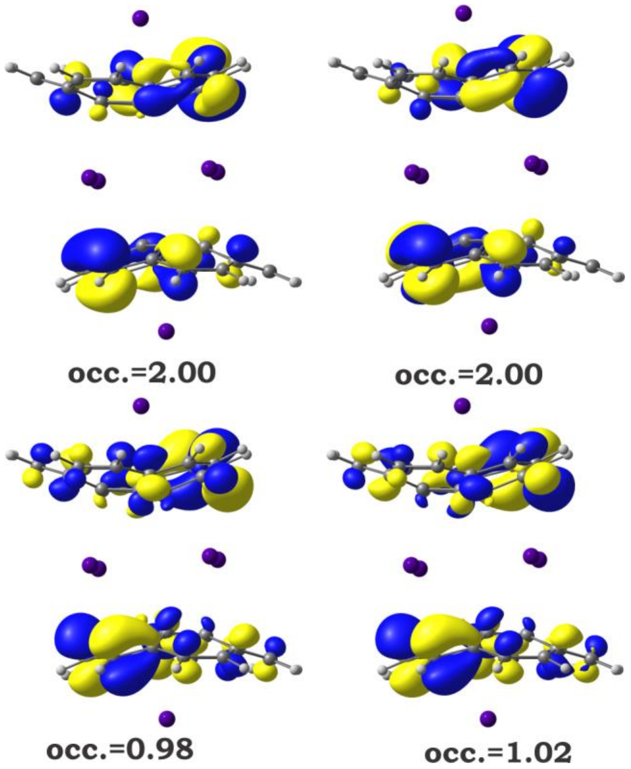

Figure S9. CASSCF(6,4) natural orbitals along with occupancies for **1H**-*small* model.

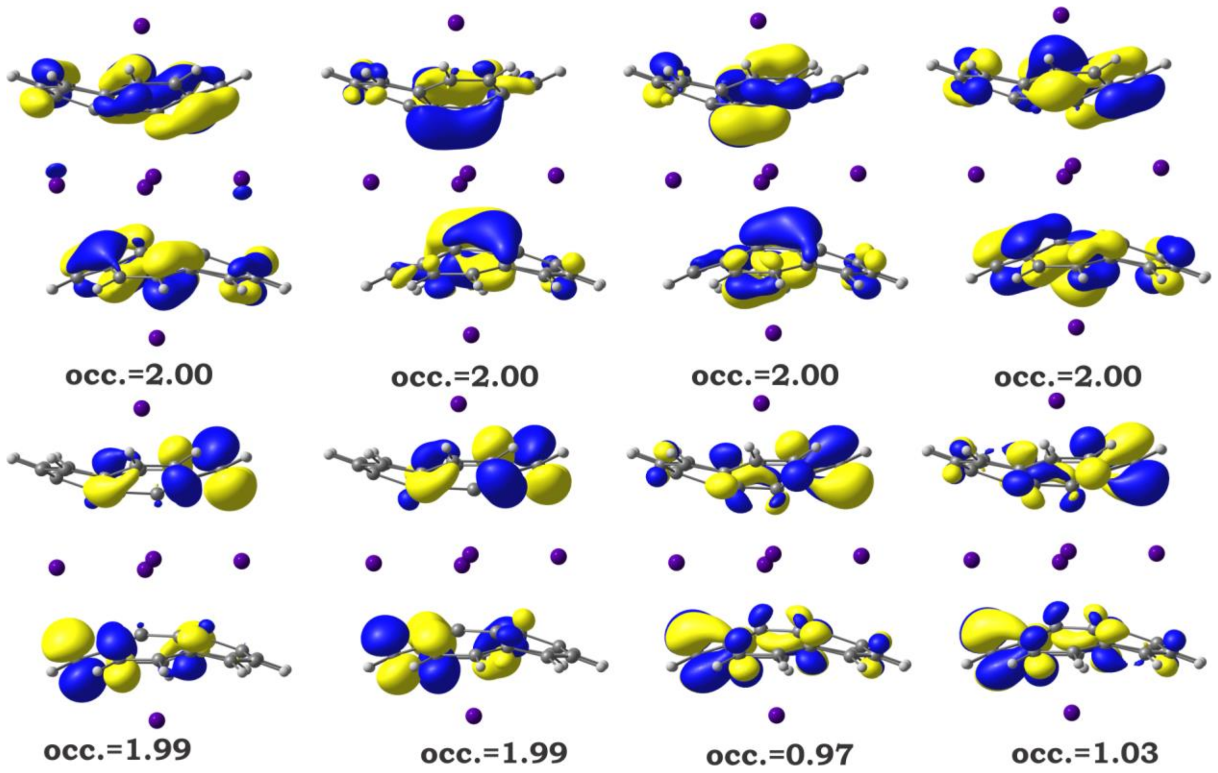

Figure S10. CASSCF(14,8) natural orbitals along with occupancies for **1H**-*small* model.

Table S2. Absolute energies of **1**-*small*, **1H**-*small*, **1**-*full*, and **1H**-*full* as well as of "naked" $C_{20}H_{10}{}^{\bullet 3-}$ anion and neutral corannulene (PBE0/def2-TZVP(Cs)//cc-pVDZ(C,H,O)).

Compound	Energy, a.u.
**1**-*small*	-1655.7565934532
**1H**-*small*	-1655.7403447424
$\mathbf{1}^{4-}$-*small*	-1675.9241888309
**1**-*full*	-4428.9866489306
**1H**-*full*	-4428.9548977736
$C_{20}H_{10}{}^{\bullet 3-}$	-766.9410257367
$C_{20}H_{10}$	-767.3150125812

Table S3. Absolute energies of all systems as results of broken-symmetry calculations (PBE0/TZVP/ZORA).

	State	Energy, a.u.
**1**-*small*	Triplet (high-spin)	-49035.059958
	Broken-symmetry	-49035.059969

**1H**-*small*	Triplet (high-spin)	-49035.045014
	Broken-symmetry	-49035.044997
**1**-*full*	Triplet (high-spin)	-51811.568480
	Broken-symmetry	-51811.568467
**1H**-*full*	Triplet (high-spin)	-51811.530492
	Broken-symmetry	-51811.530490

Table S4. Absolute energies of **1**-*small* and **1H**-*small* systems as results of MRMP2 calculations based on different converged CASSCF wavefunctions (CAS(6,4) and CAS(14,8)).

		State	Energy, a.u.
**1**-*small*	CAS(6,4)	Singlet state (OS)	-1650.579469309603
		Triplet state	-1650.579466511464
	CAS(14,8)	Singlet state (OS)	-1650.568198642138
		Triplet state	-1650.568201337413
**1H**-*small*	CAS(6,4)	Singlet state (OS)	-1650.557621718485
		Triplet state	-1650.557607114330
	CAS(14,8)	Singlet state (OS)	-1650.546739878118
		Triplet state	-1650.546721943810

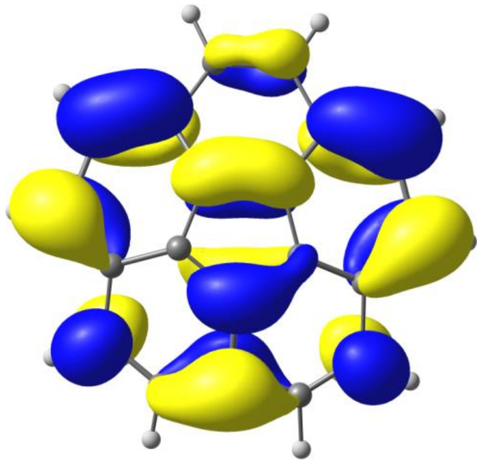

Figure S11. LUMO+1 for neutral $C_{20}H_{10}$ molecule (PBE0/cc-pVDZ).

Table S5. Cartesian coordinates for **1**-*small* system, optimized at the PBE0/def2-TZVP(Cs)//cc-pVDZ(C,H,O) level of theory.

Cs	5.293312240	0.041387413	-0.354440048
Cs	-0.117419400	-2.762787425	-1.883212717
C	3.223404160	3.335688316	-0.198425198
H	3.562490767	4.310830343	-0.562730510
C	3.400349550	3.060869815	1.184146788
H	3.836969894	3.856323907	1.800219550
C	3.132829340	1.785066810	1.770845916
C	3.541138300	1.166011648	2.990473409
H	3.983396346	1.780470849	3.783182482
C	3.529761250	-0.239801482	3.189910002
H	3.973003320	-0.620255774	4.116050687
C	3.161312350	-1.169434765	2.149382237
C	3.429794986	-2.556000974	1.944137564
H	3.890322831	-3.142757626	2.746284072
C	3.302704532	-3.185531943	0.658327757
H	3.685391873	-4.207703747	0.564024987
C	2.891437194	-2.484779812	-0.505928392
C	3.122508581	-2.712126324	-1.912033927
H	3.490026222	-3.690950105	-2.242645193
C	3.008986573	-1.696940407	-2.896657424
H	3.315168090	-1.953100494	-3.916916653
C	2.710665419	-0.312085836	-2.579533368
C	3.022422488	0.898879744	-3.243613746
H	3.371003482	0.871283084	-4.281811191
C	3.104393847	2.174870837	-2.553860171
H	3.496757082	3.025651218	-3.121535835
C	2.864399742	2.309600953	-1.160862203
C	2.398701913	1.113307263	-0.536023918
C	2.530793478	0.863656794	0.846764541
C	2.555756562	-0.535207763	1.028471413
C	2.415900585	-1.157222045	-0.240454758
C	2.325099468	-0.140977115	-1.209160726
Cs	0.114231952	-1.970148507	2.711431156
Cs	-5.293326816	-0.041380625	0.354227002
Cs	0.117448903	2.762492176	1.883772701
C	-3.223492322	-3.335604249	0.198463727
H	-3.562723309	-4.310643339	0.562751499
C	-3.400379643	-3.060861677	-1.184162613
H	-3.836998725	-3.856226093	-1.800319618
C	-3.132867460	-1.785039590	-1.770814615
C	-3.541098973	-1.165995743	-2.990566457
H	-3.983347408	-1.780379942	-3.783274131
C	-3.529614879	0.239755747	-3.190071348
H	-3.972932894	0.620244591	-4.116221538
C	-3.161315622	1.169412957	-2.149428192
C	-3.429940335	2.555960284	-1.944168921
H	-3.890292734	3.142756374	-2.746428355
C	-3.302658531	3.185500990	-0.658299108
H	-3.685637303	4.207572795	-0.563907123
C	-2.891501788	2.484694479	0.505942037
C	-3.122472697	2.712100682	1.912091291
H	-3.490187330	3.690929951	2.242530104
C	-3.009012021	1.696874093	2.896689510
H	-3.315103809	1.953197244	3.916888308
C	-2.710560297	0.312017996	2.579626926
C	-3.022433415	-0.898909292	3.243577757
H	-3.370854484	-0.871364818	4.281853238
C	-3.104236712	-2.174805809	2.553886500
H	-3.496712700	-3.025551255	3.121507779
C	-2.864427033	-2.309557334	1.160798087
C	-2.398721866	-1.113281900	0.535994330
C	-2.530724297	-0.863661485	-0.846792296
C	-2.555614623	0.535200961	-1.028533217
C	-2.415994976	1.157176439	0.240478050
C	-2.325051614	0.140911779	1.209149478
Cs	-0.114227306	1.970376764	-2.711047376

Table S6. Cartesian coordinates for **1**$^{4-}$-*small* system, optimized at the PBE0/def2-TZVP(Cs)//cc-pVDZ(C,H,O) level of theory.

Cs	-1.038500000	3.098199999	-0.679900000
Cs	1.546899999	-2.881299999	-1.332499999
C	-1.662299999	-2.049199999	-0.351900000
C	-2.287099999	-1.161599999	0.561400000
C	-2.568299999	0.047300000	-0.125200000
C	-2.117699999	-0.093100000	-1.462799999
C	-1.557899999	-1.388899999	-1.602999999
C	-1.481699999	-2.089099999	-2.866899999
C	-1.349399999	-3.505999998	-2.713999999
H	-1.323099999	-4.138299998	-3.610499998
C	-1.454099999	-4.175899998	-1.444899999
H	-1.501199999	-5.271999998	-1.462499999
C	-1.702999999	-3.488799998	-0.213900000
C	-2.284399999	-3.915999998	1.022300000
H	-2.400799999	-4.990199998	1.213199999
C	-2.916299999	-3.015399999	1.949499999
H	-3.470499998	-3.465999998	2.782499999
C	-3.025699999	-1.606399999	1.722999999
C	-3.947399998	-0.625000000	2.208799999
H	-4.588199998	-0.873000000	3.064299999
C	-4.233699998	0.600700000	1.511799999
H	-5.072699998	1.201599999	1.884699999
C	-3.622999998	0.956500000	0.267300000
C	-4.043199998	1.817799999	-0.796100000
H	-4.866499998	2.521099999	-0.618700000
C	-3.586699998	1.674999999	-2.153099999
H	-4.094199998	2.279199999	-2.915599999
C	-2.668699999	0.658900000	-2.569499999
C	-2.435599999	0.037900000	-3.838199998
H	-2.845999999	0.503800000	-4.742899998
C	-1.867699999	-1.276499999	-3.980299998
H	-1.885199999	-1.720799999	-4.983499998
Cs	0.265900000	-2.481999999	2.480699999
C	2.488399999	-0.383800000	0.868000000
C	2.591899999	0.278600000	-0.382100000
C	2.034099999	1.574599999	-0.238800000
C	1.585499999	1.713599999	1.099600000
C	1.866299999	0.503400000	1.783699999
C	2.051799999	0.425900000	3.216499999
C	2.782999999	-0.735300000	3.623499998
H	3.015399999	-0.873100000	4.687099998
C	3.414999998	-1.634499999	2.694899999
H	4.085199998	-2.394799999	3.115299999
C	3.371999998	-1.454199999	1.275599999
C	4.242599998	-1.890499999	0.226300000
H	4.982599998	-2.672499999	0.438400000
C	4.346999998	-1.219199999	-1.042200000
H	5.159299998	-1.536399999	-1.708199999
C	3.590499998	-0.050900000	-1.376099999
C	3.826199998	1.012400000	-2.304999999
H	4.593699998	0.883800000	-3.078599999
C	3.258399999	2.326599999	-2.160699999
H	3.632199998	3.107799999	-2.834399999
C	2.403999999	2.696599999	-1.073300000
C	2.108899999	3.961699998	-0.472200000
H	2.385899999	4.881299998	-1.002900000
C	1.654099999	4.102799998	0.885400000
H	1.615899999	5.119999998	1.294999999
C	1.453899999	2.991499999	1.764999999
C	1.466299999	2.881999999	3.192199999
H	1.412399999	3.795899998	3.797099998
C	1.752299999	1.654799999	3.886499998
H	1.897399999	1.718899999	4.972199998
Cs	-4.540699998	-1.926899999	-1.342599999
Cs	4.620399998	1.729699999	1.362699999
Cs	0.741100000	0.569100000	-3.280599999
Cs	-1.327099999	1.211499999	2.880599999

Table S7. Cartesian coordinates for **1H**-*small* system, optimized at the PBE0/def2-TZVP(Cs)//cc-pVDZ(C,H,O) level of theory.

Cs	5.293300000	0.041400000	-0.354400000
Cs	-0.117400000	-2.762800000	-1.883200000
C	3.223400000	3.335700000	-0.198400000
H	3.562500000	4.310800000	-0.562700000
C	3.400300000	3.060900000	1.184100000
H	3.837000000	3.856300000	1.800200000
C	3.132800000	1.785100000	1.770800000
C	3.541100000	1.166000000	2.990500000
H	3.983400000	1.780500000	3.783200000
C	3.529800000	-0.239800000	3.189900000
H	3.973000000	-0.620300000	4.116100000
C	3.161300000	-1.169400000	2.149400000
C	3.429800000	-2.556000000	1.944100000
H	3.890300000	-3.142800000	2.746300000
C	3.302700000	-3.185500000	0.658300000
H	3.685400000	-4.207700000	0.564000000
C	2.891400000	-2.484800000	-0.505900000
C	3.122500000	-2.712100000	-1.912000000
H	3.490000000	-3.691000000	-2.242600000
C	3.009000000	-1.696900000	-2.896700000
H	3.315200000	-1.953100000	-3.916900000
C	2.710700000	-0.312100000	-2.579500000
C	3.022400000	0.898900000	-3.243600000
H	3.371000000	0.871300000	-4.281800000
C	3.104400000	2.174900000	-2.553900000
H	3.496800000	3.025700000	-3.121500000
C	2.864400000	2.309600000	-1.160900000
C	2.398700000	1.113300000	-0.536000000
C	2.530800000	0.863700000	0.846800000
C	2.555800000	-0.535200000	1.028500000
C	2.415900000	-1.157200000	-0.240500000
C	2.325100000	-0.141000000	-1.209200000
Cs	0.114200000	-1.970100000	2.711400000
Cs	-5.293300000	-0.041400000	0.354200000
Cs	0.117400000	2.762500000	1.883800000
C	-3.223500000	-3.335600000	0.198500000
H	-3.562700000	-4.310600000	0.562800000
C	-3.400400000	-3.060900000	-1.184200000
H	-3.837000000	-3.856200000	-1.800300000
C	-3.132900000	-1.785000000	-1.770800000
C	-3.541100000	-1.166000000	-2.990600000
H	-3.983300000	-1.780400000	-3.783300000
C	-3.529600000	0.239800000	-3.190100000
H	-3.972900000	0.620200000	-4.116200000
C	-3.161300000	1.169400000	-2.149400000
C	-3.429900000	2.556000000	-1.944200000
H	-3.890300000	3.142800000	-2.746400000
C	-3.302700000	3.185500000	-0.658300000
H	-3.685600000	4.207600000	-0.563900000
C	-2.891500000	2.484700000	0.505900000
C	-3.122500000	2.712100000	1.912100000
H	-3.490200000	3.690900000	2.242500000
C	-3.009000000	1.696900000	2.896700000
H	-3.315100000	1.953200000	3.916900000
C	-2.710600000	0.312000000	2.579600000
C	-3.022400000	-0.898900000	3.243600000
H	-3.370900000	-0.871400000	4.281900000
C	-3.104200000	-2.174800000	2.553900000
H	-3.496700000	-3.025600000	3.121500000
C	-2.864400000	-2.309600000	1.160800000
C	-2.398700000	-1.113300000	0.536000000
C	-2.530700000	-0.863700000	-0.846800000
C	-2.555600000	0.535200000	-1.028500000
C	-2.416000000	1.157200000	0.240500000
C	-2.325100000	0.140900000	1.209100000
Cs	-0.114200000	1.970400000	-2.711000000

Table S8. Cartesian coordinates for **1**-*full* system, optimized at the PBE0/def2-TZVP(Cs)//cc-pVDZ(C,H,O) level of theory.

Cs	-5.296100000	0.191800000	-1.545500000
Cs	5.296000000	-0.191800000	1.545500000
Cs	-0.492800000	2.422200000	1.986800000
Cs	0.492800000	-2.422200000	-1.986800000
O	-7.537600000	2.554100000	-0.933200000
O	7.537600000	-2.554100000	0.933200000
O	-7.899100000	-0.079600000	0.093400000
O	7.899100000	0.079600000	-0.093400000
O	-7.539500000	-2.195200000	-1.778800000
O	7.539500000	2.195200000	1.778800000
O	-1.793900000	4.015800000	4.858700000
O	1.793800000	-4.015800000	-4.858700000
O	-0.498800000	5.501200000	2.759400000
O	0.498800000	-5.501200000	-2.759400000
O	0.252700000	5.077800000	0.100800000
O	-0.252700000	-5.077800000	-0.100800000
C	-3.228900000	3.261700000	-0.153800000
C	3.228900000	-3.261600000	0.153800000
H	-3.424800000	4.286800000	-0.489200000
H	3.424800000	-4.286900000	0.489200000
C	-3.782600000	2.873500000	1.128200000
C	3.782600000	-2.873500000	-1.128200000
H	-4.355300000	3.632200000	1.674900000
H	4.355300000	-3.632200000	-1.675000000
C	-3.767500000	1.531000000	1.587200000
C	3.767500000	-1.531000000	-1.587200000
C	-4.525500000	0.809800000	2.591500000
C	4.525500000	-0.809800000	-2.591600000
H	-5.136500000	1.372900000	3.307400000
H	5.136500000	-1.372900000	-3.307400000
C	-4.652900000	-0.596700000	2.603000000
C	4.652900000	0.596800000	-2.603100000
H	-5.340900000	-1.032400000	3.337200000
H	5.340900000	1.032300000	-3.337100000
C	-4.031000000	-1.453600000	1.629800000
C	4.031000000	1.453600000	-1.629900000
C	-4.285700000	-2.776400000	1.186100000
C	4.285700000	2.776400000	-1.186100000
H	-4.947700000	-3.427400000	1.770500000
H	4.947700000	3.427300000	-1.770500000
C	-3.806600000	-3.283100000	-0.071800000
C	3.806600000	3.283100000	0.071800000
H	-4.166600000	-4.272200000	-0.380200000
H	4.166600000	4.272200000	0.380200000
C	-3.075000000	-2.474200000	-1.013700000
C	3.074900000	2.474200000	1.013700000
C	2.875400000	2.559300000	2.432300000
C	-2.875400000	-2.559300000	-2.432300000
H	3.202900000	3.453600000	2.975200000
H	-3.203000000	-3.453600000	-2.975100000
C	2.478000000	1.422200000	3.228100000
C	-2.478100000	-1.422200000	-3.228200000
H	2.517400000	1.540700000	4.317900000
H	-2.517500000	-1.540800000	-4.317900000
C	2.181100000	0.149100000	2.675000000
C	-2.181100000	-0.149100000	-2.675000000
C	2.205100000	-1.174600000	3.243700000
C	-2.205100000	1.174600000	-3.243700000
H	2.198700000	-1.279600000	4.335500000
H	-2.198700000	1.279600000	-4.335500000
C	2.401300000	-2.355200000	2.477000000
C	-2.401300000	2.355100000	-2.477000000
H	2.554100000	-3.291800000	3.029200000
H	-2.554100000	3.291800000	-3.029200000
C	-2.618900000	2.339300000	-1.043600000
C	2.618900000	-2.339300000	1.043600000
C	-2.426700000	1.038900000	-0.484400000
C	2.426700000	-1.039000000	0.484400000
C	-3.008500000	0.657000000	0.757900000
C	3.008500000	-0.657000000	-0.757900000
C	-3.138300000	-0.737100000	0.770400000

C	3.138300000	0.737100000	-0.770400000
C	-2.657100000	-1.228400000	-0.466300000
C	2.657100000	1.228400000	0.466300000
C	2.202000000	0.127600000	1.238100000
C	-2.202000000	-0.127600000	-1.238100000
C	-6.888200000	3.791300000	-1.123700000
C	6.888200000	-3.791300000	1.123700000
H	-5.888500000	3.803700000	-0.652400000
H	5.888500000	-3.803700000	0.652400000
H	-6.777500000	3.938100000	-2.207300000
H	6.777500000	-3.938000000	2.207400000
H	-7.492700000	4.624600000	-0.713900000
H	7.492700000	-4.624600000	0.714000000
C	-7.752800000	2.274300000	0.432700000
C	7.752800000	-2.274300000	-0.432700000
H	-6.790500000	2.131400000	0.964300000
H	6.790400000	-2.131400000	-0.964300000
H	-8.286000000	3.118200000	0.916600000
H	8.286000000	-3.118200000	-0.916600000
C	-8.609800000	1.041700000	0.557100000
C	8.609800000	-1.041700000	-0.557200000
H	-9.539400000	1.176700000	-0.031300000
H	9.539400000	-1.176700000	0.031300000
H	-8.893400000	0.907800000	1.620500000
H	8.893300000	-0.907800000	-1.620500000
C	-8.666400000	-1.258000000	0.098600000
C	8.666400000	1.258000000	-0.098600000
H	-9.003500000	-1.496300000	1.127700000
H	9.003400000	1.496300000	-1.127700000
H	-9.566700000	-1.132000000	-0.535600000
H	9.566700000	1.132000000	0.535500000
C	-7.837100000	-2.405300000	-0.415800000
C	7.837100000	2.405300000	0.415700000
H	-8.421700000	-3.340100000	-0.291600000
H	8.421700000	3.340100000	0.291600000
H	-6.906400000	-2.502600000	0.178300000
H	6.906400000	2.502600000	-0.178300000
C	-6.884100000	-3.305900000	-2.350200000
C	6.884200000	3.305900000	2.350100000
H	-7.527300000	-4.207500000	-2.315100000
H	7.527300000	4.207500000	2.315000000
H	-6.677200000	-3.060500000	-3.400900000
H	6.677300000	3.060500000	3.400900000
H	-5.931200000	-3.522500000	-1.834200000
H	5.931200000	3.522500000	1.834200000
C	-2.793300000	3.146900000	5.339300000
C	2.793300000	-3.146800000	-5.339300000
H	-3.494900000	3.677600000	6.012800000
H	3.494900000	-3.677600000	-6.012800000
H	-2.290500000	2.356900000	5.912200000
H	2.290400000	-2.356900000	-5.912200000
H	-3.367400000	2.685300000	4.514100000
H	3.367400000	-2.685400000	-4.514200000
C	-2.347700000	5.139100000	4.219200000
C	2.347600000	-5.139100000	-4.219200000
H	-3.025700000	5.679200000	4.912500000
H	3.025600000	-5.679200000	-4.912500000
H	-2.938300000	4.831700000	3.332700000
H	2.938300000	-4.831700000	-3.332700000
C	-1.250900000	6.080500000	3.797600000
C	1.250900000	-6.080500000	-3.797600000
H	-1.715000000	7.026000000	3.450200000
H	1.715000000	-7.026000000	-3.450200000
H	-0.602300000	6.318100000	4.664000000
H	0.602200000	-6.318100000	-4.664000000
C	0.407600000	6.397400000	2.169200000
C	-0.407600000	-6.397400000	-2.169100000
H	1.172700000	6.737200000	2.896900000
H	-1.172700000	-6.737300000	-2.896800000
H	-0.132800000	7.302300000	1.823000000
H	0.132900000	-7.302300000	-1.823000000
C	1.118200000	5.705400000	1.022500000
C	-1.118200000	-5.705400000	-1.022500000
H	-1.760000000	-6.452500000	-0.513200000
H	1.760000000	6.452500000	0.513300000
H	1.764500000	4.899500000	1.403400000
H	-1.764500000	-4.899500000	-1.403300000

C	0.644100000	-5.948700000	0.547200000
C	-0.644100000	5.948700000	-0.547200000
H	-1.386500000	6.372100000	0.151400000
H	1.386500000	-6.372100000	-0.151400000
H	1.187200000	-5.349100000	1.290100000
H	-1.187200000	5.349200000	-1.290100000
H	0.105200000	-6.765700000	1.062500000
H	-0.105200000	6.765800000	-1.062500000
Cs	-1.005000000	-2.783500000	2.137000000
Cs	1.005000000	2.783500000	-2.137000000
O	-1.719800000	-5.801900000	2.987200000
O	1.719900000	5.801900000	-2.987100000
O	-0.837100000	-4.399600000	5.264400000
O	0.837100000	4.399700000	-5.264300000
O	-1.174100000	-1.654700000	5.015400000
O	1.174100000	1.654700000	-5.015400000
C	-2.868100000	-6.358600000	2.396400000
C	2.868100000	6.358600000	-2.396400000
H	-3.726700000	-6.340800000	3.093900000
H	3.726700000	6.340800000	-3.093900000
H	-3.112000000	-5.743000000	1.519200000
H	3.112000000	5.743000000	-1.519100000
H	-2.694600000	-7.406400000	2.080100000
H	2.694700000	7.406400000	-2.080100000
C	-1.354500000	-6.435000000	4.187000000
C	1.354600000	6.435000000	-4.187000000
H	-2.228800000	-6.515900000	4.862500000
H	2.228800000	6.515900000	-4.862500000
H	-0.977500000	-7.460400000	3.993700000
H	0.977600000	7.460500000	-3.993700000
C	-0.279000000	-5.622800000	4.862000000
C	0.279000000	5.622800000	-4.862000000
H	0.564300000	-5.459600000	4.159800000
H	-0.564200000	5.459600000	-4.159800000
H	0.115300000	-6.188100000	5.731500000
H	-0.115300000	6.188100000	-5.731500000
C	0.104300000	-3.497400000	5.795000000
C	-0.104300000	3.497500000	-5.795000000
H	0.588100000	-3.916400000	6.701100000
H	-0.588100000	3.916500000	-6.701100000
H	0.898300000	-3.284100000	5.052300000
H	-0.898300000	3.284100000	-5.052300000
C	-0.605100000	-2.220100000	6.166000000
C	0.605100000	2.220100000	-6.166000000
H	0.128900000	-1.527700000	6.626200000
H	-0.128900000	1.527800000	-6.626100000
H	-1.391600000	-2.432400000	6.918400000
H	1.391500000	2.432400000	-6.918400000
C	-1.825800000	-0.434700000	5.269400000
C	1.825700000	0.434700000	-5.269300000
H	-1.100900000	0.346700000	5.569500000
H	1.100800000	-0.346700000	-5.569500000
H	-2.344800000	-0.131000000	4.348000000
H	2.344800000	0.131000000	-4.347900000
H	-2.583100000	-0.543400000	6.069900000
H	2.583000000	0.543300000	-6.069800000

Table S9. Cartesian coordinates for **1H-*full*** system, optimized at the PBE0/def2-TZVP(Cs)//cc-pVDZ(C,H,O) level of theory.

Cs	5.229041342	-0.077637756	1.649744011
Cs	-0.617790374	2.486193422	2.107884411
O	7.624645400	-2.318698620	0.908430134
O	7.611308755	0.133960601	-0.277981528
O	7.748973801	2.105305785	1.542894653
O	-2.140112765	3.892351450	4.762827726
O	-0.493534002	5.436745582	2.941294647
O	0.309239866	4.967563509	0.319014051
C	3.406490518	-3.458906122	0.045315138
H	3.643070826	-4.495868702	0.330365659
C	3.934989695	-2.985821201	-1.196844868
H	4.542354415	-3.691800226	-1.786196082

C	3.849828287	-1.624241291	-1.595714081
C	4.620329719	-0.825642969	-2.493791601
H	5.299921385	-1.323303690	-3.205643558
C	4.673396876	0.589597594	-2.446384620
H	5.379312230	1.099328456	-3.119248668
C	3.950121941	1.357352447	-1.467473589
C	4.107910390	2.684883887	-0.968936351
H	4.761575380	3.387399522	-1.508758443
C	3.601893861	3.094125700	0.313073406
H	3.942451869	4.068018182	0.702482429
C	2.847132952	2.236365869	1.158624015
C	2.652022637	2.232837585	2.583102378
H	2.917848641	3.128684341	3.168550321
C	2.301626478	1.060507095	3.318901365
H	2.322731162	1.128333233	4.417941939
C	2.129305089	-0.220979303	2.700560363
C	2.337187439	-1.529050757	3.230163432
H	2.440888466	-1.638412785	4.321217447
C	2.620897778	-2.679173973	2.424433651
H	2.911591294	-3.606888340	2.942513897
C	2.762281316	-2.600064300	0.997198015
C	2.472524520	-1.302052128	0.482756956
C	3.020875973	-0.826880976	-0.724218547
C	3.042649164	0.573715420	-0.699528673
C	2.487126502	0.988602394	0.541754426
C	2.137285106	-0.182760792	1.282259046
C	6.962513681	-3.455279687	1.463015062
H	5.874808345	-3.454465242	1.233163444
H	7.110853082	-3.419705954	2.555320920
H	7.396166585	-4.405228618	1.078841585
C	7.431696274	-2.206359311	-0.492592449
H	6.349394719	-2.150887299	-0.743255935
H	7.844940072	-3.097293785	-1.022966898
C	8.141830223	-0.983223975	-0.969755469
H	9.235769958	-1.066288149	-0.773497694
H	7.994719173	-0.881090805	-2.068720206
C	8.224518678	1.358953484	-0.664537313
H	8.054114437	1.574579345	-1.743166540
H	9.322899675	1.302661074	-0.488991360
C	7.609755021	2.451922689	0.168674320
H	8.125247576	3.415576447	-0.052043970
H	6.534091955	2.575414995	-0.094558946
C	7.257506710	3.118888756	2.406231807
H	7.835403064	4.064445662	2.303740012
H	7.365692433	2.754466739	3.441720161
H	6.186604119	3.345409292	2.208827225
C	-3.353960481	3.238341594	5.108231835
H	-3.905103717	3.806861692	5.889680550
H	-3.102016620	2.246294492	5.510602206
H	-4.008242500	3.104672508	4.224354686
C	-2.411732670	5.219258123	4.324124214
H	-2.998758865	5.761315098	5.102213127
H	-3.021568591	5.202597748	3.392689617
C	-1.146525185	5.951526008	4.089053574
H	-1.391610217	7.027262081	3.935424644
H	-0.482313746	5.885633383	4.979752808
C	0.443885767	6.323578729	2.376834449
H	1.207133975	6.647632116	3.121638004
H	-0.071327618	7.247417288	2.023736456
C	1.165541514	5.613467478	1.248353777
H	1.824458811	6.350221282	0.735999722
H	1.808323076	4.808946017	1.647312986
C	-0.598502704	5.852029893	-0.342844211
H	-1.334416409	6.286928628	0.360514377
H	-1.150595305	5.249295269	-1.082007012
H	-0.053166024	6.667487574	-0.863091565
Cs	0.670043342	2.503344322	-1.870518542
Cs	-0.669983986	-2.503342506	1.870515488
Cs	0.617849729	-2.486191605	-2.107887467
C	-3.406431163	3.458907939	-0.045318192
C	-3.934930342	2.985823018	1.196841815
C	-3.849768933	1.624243114	1.595711031
C	-2.472465163	1.302053945	-0.482760011
C	-3.020816842	0.826883084	0.724214564
O	1.674327647	5.295559265	-2.841238122
O	0.869156993	3.898137363	-5.037188949
O	1.250344411	1.257876503	-4.713982997

C	2.650051903	6.027850074	-2.099947537
H	3.515514789	6.303750299	-2.739198902
H	2.998232987	5.366708124	-1.290278364
H	2.220599786	6.961513037	-1.672756372
C	1.246427370	5.983632424	-4.005785434
H	2.116547740	6.189651691	-4.669669884
H	0.782208698	6.964425940	-3.747210955
C	0.248675538	5.131300262	-4.717161582
H	-0.643249098	4.958794581	-4.069966424
H	-0.104259402	5.662370881	-5.632382365
C	-0.003796055	3.008010949	-5.723983828
H	-0.336220518	3.434004738	-6.699047954
H	-0.904040799	2.803932513	-5.106268022
C	0.754776362	1.729641025	-5.962925535
H	0.077314374	0.982387714	-6.435751880
H	1.604613082	1.908789938	-6.660509045
C	1.921097549	0.013153876	-4.836446200
H	1.211458585	-0.806975556	-5.086119235
H	2.436324806	-0.192623734	-3.881864037
H	2.699761603	0.057160818	-5.629104129
C	-2.129245732	0.220981119	-2.700563417
C	-2.337128082	1.529052571	-3.230166485
C	-2.620838422	2.679175787	-2.424436704
C	-2.762221958	2.600066115	-0.997201071
C	-2.137225749	0.182762608	-1.282262100
Cs	-5.228981985	0.077639572	-1.649747066
O	-7.624586029	2.318700422	-0.908433190
O	-7.611249395	-0.133958787	0.277978471
O	-7.748914651	-2.105303663	-1.542898639
O	2.140172125	-3.892349635	-4.762830798
O	0.493593355	-5.436743767	-2.941297704
O	-0.309180511	-4.967561690	-0.319017105
H	-3.643696612	4.495676299	-0.330501253
H	-4.543101016	3.691582993	1.785630958
C	-4.620270355	0.825644805	2.493788550
H	-5.300048584	1.323374856	3.205407630
C	-4.673337507	-0.589595798	2.446381562
H	-5.379801180	-1.099325277	3.118675149
C	-3.950062583	-1.357350632	1.467470537
C	-4.107851034	-2.684882071	0.968933297
H	-4.761723781	-3.387305119	1.508628075
C	-3.601834506	-3.094123885	-0.313076459
H	-3.943150324	-4.067546578	-0.702992840
C	-2.847073597	-2.236364051	-1.158627069
C	-2.651963282	-2.232835768	-2.583105433
H	-2.918111393	-3.128580766	-3.168567582
C	-2.301567121	-1.060505278	-3.318904419
H	-2.322367890	-1.128311568	-4.417950776
H	-2.440485049	1.638540735	-4.321234729
H	-2.912934268	3.606408967	-2.942587152
C	-3.042589806	-0.573713604	0.699525620
C	-2.487067145	-0.988600577	-0.541757479
C	-6.962454325	3.455281508	-1.463018116
H	-5.873257497	3.450032677	-1.240396524
H	-7.118902181	3.425491415	-2.554314380
H	-7.390179928	4.404651088	-1.070842996
C	-7.431636924	2.206361124	0.492589401
H	-6.349336506	2.150595568	0.743292723
H	-7.844572097	3.097593751	1.022691708
C	-8.141770859	0.983225771	0.969752405
H	-9.235709718	1.066282113	0.773466106
H	-7.994705058	0.881076464	2.068718313
C	-8.224459317	-1.358951639	0.664534252
H	-8.054265162	-1.574524625	1.743204761
H	-9.322840154	-1.302643866	0.488915069
C	-7.609695670	-2.451920873	-0.168677375
H	-8.125184324	-3.415630142	0.051751409
H	-6.534080441	-2.575477990	0.094735802
C	-7.257447364	-3.118886947	-2.406234860
H	-7.829021521	-4.067492385	-2.296522072
H	-7.375243406	-2.758689692	-3.442143491
H	-6.183998322	-3.338999604	-2.215440548
C	3.354019846	-3.238339763	-5.108234883
H	3.909094003	-3.810853248	-5.883930664
H	3.101587335	-2.249490059	-5.518263686
H	4.004910535	-3.097076830	-4.223024142
C	2.411792022	-5.219256303	-4.324127272

H	2.998995127	-5.761241637	-5.102095289
H	3.021290918	-5.202687326	-3.392461313
C	1.146584539	-5.951524186	-4.089056636
H	1.391593121	-7.027286941	-3.935549482
H	0.482336050	-5.885486533	-4.979731160
C	-0.443826412	-6.323576912	-2.376837510
H	-1.207091995	-6.647621988	-3.121630252
H	0.071390484	-7.247434396	-2.023787153
C	-1.165482159	-5.613465664	-1.248356834
H	-1.824231782	-6.350392925	-0.736039501
H	-1.808341504	-4.809044152	-1.647386576
C	0.598562060	-5.852028075	0.342841152
H	1.334354432	-6.287339612	-0.360375670
H	1.150548028	-5.249177756	1.081974849
H	0.053137336	-6.667353930	0.863237822
O	-1.674268292	-5.295557449	2.841235064
O	-0.869097638	-3.898135552	5.037185896
O	-1.250285054	-1.257874688	4.713979946
C	-2.649992548	-6.027848262	2.099944482
H	-3.515346191	-6.303526458	2.739442688
H	-2.998420244	-5.366588676	1.290466792
H	-2.220659051	-6.961463010	1.672573595
C	-1.246368012	-5.983630605	4.005782376
H	-2.116494456	-6.189644101	4.669663924
H	-0.782147095	-6.964425202	3.747224087
C	-0.248616181	-5.131298450	4.717158527
H	0.643309952	-4.958805579	4.069964114
H	0.104333010	-5.662366227	5.632377095
C	0.003855411	-3.008009136	5.723980769
H	0.336117937	-3.434061583	6.699079990
H	0.904190858	-2.803990385	5.106378239
C	-0.754717004	-1.729639211	5.962922488
H	-0.077154517	-0.982469951	6.435743348
H	-1.604364280	-1.908865898	6.660707599
C	-1.921038197	-0.013152058	4.836443147
H	-1.210548519	0.808003131	5.080265187
H	-2.441345114	0.189548400	3.883914953
H	-2.695349768	-0.055266861	5.633459395

Table S10. Cartesian coordinates for "naked" trianion $C_{20}H_{10}^{\bullet 3-}$, optimized at the PBE0/cc-pVDZ level of theory.

C	-3.265015562	0.235658907	0.720595975
C	-1.702839165	0.239717050	-2.869905617
C	2.225304702	0.227635818	-2.493165641
C	3.066918203	0.243604498	1.322069513
C	-0.325119872	0.230639008	3.321121380
H	-4.214864304	0.508394695	1.215960021
H	-2.466743225	0.547506396	-3.606190104
H	2.699121969	0.505222477	-3.451713977
H	4.122522033	0.533658823	1.475711385
H	-0.140462667	0.530425700	4.367842704
C	-2.073693736	0.017338442	-1.515605622
C	0.786318393	0.019107786	-2.441776288
C	2.583438882	0.009444633	0.003397766
C	0.789490813	0.021649954	2.432666782
C	-2.085616106	0.010385502	1.521130559
C	-0.973298443	-0.344393466	-0.694682545
C	0.377699884	-0.341837798	-1.132940328
C	1.201337094	-0.352061348	-0.005224989
C	0.368825595	-0.337452829	1.131483121
C	-0.975809337	-0.352078037	0.699905312
C	-3.266469277	0.228211803	-0.710013059
C	-0.311371997	0.238063347	-3.322343825
C	3.065827335	0.237776284	-1.333238275
C	2.217904604	0.228999260	2.494108845
C	-1.704874197	0.244219829	2.870841615
H	-4.214523117	0.502915166	-1.206513780
H	-0.125689077	0.546673114	-4.366559076
H	4.125056646	0.512314509	-1.490582498
H	2.685181410	0.524348416	3.450073961
H	-2.468557480	0.538825057	3.613544678

Table S11. Cartesian coordinates for neutral $C_{20}H_{10}$, optimized at the PBE0/cc-pVDZ level of theory.

C	-3.209723411	0.253124867	0.698625151
C	-1.661468844	0.291317307	-2.808774635
C	2.191967488	0.254550867	-2.441317888
C	3.004107163	0.275987225	1.300290667
C	-0.325275676	0.277394691	3.251477159
H	-4.120810974	0.586604390	1.209142910
H	-2.422914522	0.677201734	-3.494695670
H	2.624557273	0.592031173	-3.389403051
H	4.030824922	0.641626936	1.415412809
H	-0.112461853	0.648111131	4.259378665
C	-2.021240707	-0.052249624	-1.477159524
C	0.766283172	-0.049102069	-2.378785023
C	2.521536553	-0.068714640	0.003582705
C	0.768637037	-0.043425260	2.368487576
C	-2.034810038	-0.066015912	1.484737381
C	-0.976033088	-0.550202178	-0.697129172
C	0.378453573	-0.547538675	-1.136147411
C	1.206915877	-0.561492273	-0.004717277
C	0.369911900	-0.542120630	1.134238199
C	-0.979628587	-0.559959318	0.703197836
C	-3.210290204	0.251922050	-0.687653835
C	-0.309637393	0.290756977	-3.248277504
C	3.007712036	0.256589923	-1.316904656
C	2.179184907	0.271906005	2.438640987
C	-1.666394046	0.281168357	2.814071481
H	-4.119841223	0.584873643	-1.199809496
H	-0.096155622	0.676265746	-4.250641498
H	4.043344352	0.595258350	-1.438469412
H	2.601796470	0.635551153	3.380889950
H	-2.428546536	0.653491052	3.507710570

Table S12. NBO charges for **1**-*small* model (PBE0/def2-TZVP(Cs)//cc-pvDZ(C,H,O)).

Atom	No	Natural Charge	Natural Population				Natural Spin Density
			Core	Valence	Rydberg	Total	
Cs	1	0.96110	53.99240	0.01210	0.03440	54.03890	0.00173
Cs	2	0.92064	53.98568	0.03767	0.05601	54.07936	0.01347
C	3	-0.36674	1.99900	4.34522	0.02253	6.36674	0.03629
H	4	0.22893	0.00000	0.76784	0.00323	0.77107	0.00020
C	5	-0.31241	1.99898	4.29228	0.02114	6.31241	0.09691
H	6	0.22853	0.00000	0.76810	0.00336	0.77147	0.00014
C	7	-0.21280	1.99879	4.19095	0.02306	6.21280	0.00295
C	8	-0.27897	1.99898	4.26030	0.01969	6.27897	0.09089
H	9	0.22641	0.00000	0.77028	0.00331	0.77359	0.00006
C	10	-0.35623	1.99899	4.33585	0.02139	6.35623	0.01911
H	11	0.22703	0.00000	0.76972	0.00325	0.77297	0.00015
C	12	-0.14253	1.99878	4.12185	0.02190	6.14253	0.07686
C	13	-0.38724	1.99899	4.36545	0.02280	6.38724	0.00272
H	14	0.22756	0.00000	0.76910	0.00333	0.77244	0.00009
C	15	-0.31320	1.99898	4.29364	0.02058	6.31320	0.06730
H	16	0.22685	0.00000	0.76988	0.00327	0.77315	0.00003
C	17	-0.17848	1.99878	4.15739	0.02231	6.17848	0.02482
C	18	-0.34941	1.99900	4.32781	0.02260	6.34941	0.12171
H	19	0.22998	0.00000	0.76656	0.00345	0.77002	0.00019
C	20	-0.35265	1.99900	4.33077	0.02289	6.35265	0.11664
H	21	0.22993	0.00000	0.76671	0.00336	0.77007	0.00017
C	22	-0.14703	1.99877	4.12694	0.02131	6.14703	0.03392
C	23	-0.37640	1.99898	4.35432	0.02310	6.37640	0.05215
H	24	0.22944	0.00000	0.76727	0.00330	0.77056	0.00008
C	25	-0.41714	1.99899	4.39390	0.02424	6.41714	0.00663
H	26	0.22987	0.00000	0.76673	0.00340	0.77013	0.00004
C	27	-0.13245	1.99877	4.11223	0.02144	6.13245	0.06488
C	28	-0.14343	1.99874	4.12353	0.02116	6.14343	0.05358

C 29	-0.17325	1.99873	4.15201	0.02251	6.17325	0.00216
C 30	-0.13338	1.99874	4.11367	0.02096	6.13338	0.05287
C 31	-0.16042	1.99873	4.13991	0.02177	6.16042	0.01987
C 32	-0.15830	1.99873	4.13819	0.02138	6.15830	0.02736
Cs 33	0.92618	53.98663	0.03178	0.05542	54.07382	0.01403
Cs 34	0.96110	53.99240	0.01211	0.03439	54.03890	0.00172
Cs 35	0.92063	53.98568	0.03767	0.05602	54.07937	0.01348
C 36	-0.36686	1.99900	4.34534	0.02253	6.36686	0.03605
H 37	0.22892	0.00000	0.76785	0.00323	0.77108	0.00020
C 38	-0.31242	1.99898	4.29230	0.02114	6.31242	0.09683
H 39	0.22853	0.00000	0.76810	0.00336	0.77147	0.00014
C 40	-0.21277	1.99879	4.19092	0.02306	6.21277	0.00293
C 41	-0.27892	1.99898	4.26025	0.01969	6.27892	0.09098
H 42	0.22641	0.00000	0.77028	0.00331	0.77359	0.00006
C 43	-0.35614	1.99899	4.33576	0.02138	6.35614	0.01927
H 44	0.22704	0.00000	0.76971	0.00325	0.77296	0.00015
C 45	-0.14256	1.99878	4.12188	0.02190	6.14256	0.07679
C 46	-0.38720	1.99899	4.36541	0.02280	6.38720	0.00274
H 47	0.22757	0.00000	0.76909	0.00333	0.77243	0.00009
C 48	-0.31337	1.99898	4.29381	0.02058	6.31337	0.06709
H 49	0.22685	0.00000	0.76988	0.00327	0.77315	0.00003
C 50	-0.17833	1.99878	4.15725	0.02230	6.17833	0.02495
C 51	-0.34957	1.99900	4.32796	0.02261	6.34957	0.12161
H 52	0.22999	0.00000	0.76655	0.00345	0.77001	0.00019
C 53	-0.35249	1.99900	4.33061	0.02288	6.35249	0.11686
H 54	0.22992	0.00000	0.76672	0.00336	0.77008	0.00017
C 55	-0.14720	1.99877	4.12711	0.02132	6.14720	0.03379
C 56	-0.37618	1.99898	4.35410	0.02310	6.37618	0.05238
H 57	0.22944	0.00000	0.76726	0.00330	0.77056	0.00008
C 58	-0.41729	1.99899	4.39404	0.02425	6.41729	0.00656
H 59	0.22987	0.00000	0.76673	0.00340	0.77013	0.00004
C 60	-0.13233	1.99877	4.11212	0.02144	6.13233	0.06499
C 61	-0.14335	1.99874	4.12346	0.02116	6.14335	0.05366
C 62	-0.17328	1.99873	4.15203	0.02251	6.17328	0.00214
C 63	-0.13343	1.99874	4.11372	0.02097	6.13343	0.05282
C 64	-0.16033	1.99873	4.13983	0.02177	6.16033	0.01998
C 65	-0.15840	1.99873	4.13828	0.02138	6.15840	0.02723
Cs 66	0.92617	53.98663	0.03179	0.05541	54.07383	0.01402
* Total *	0.00000	403.88436	184.87989	1.23575	590.00000	2.00000

Table S13. NBO charges for **1**-*small* model (PBE0/def2-TZVP(Cs)//cc-pvDZ(C,H,O)).

Atom No	Natural Charge	Natural Population Core	Valence	Rydberg	Total	Natural Spin Density
Cs 1	0.96110	53.99240	0.01210	0.03440	54.03890	0.00173
Cs 2	0.92064	53.98568	0.03767	0.05601	54.07936	0.01347
C 3	-0.36674	1.99900	4.34522	0.02253	6.36674	0.03629
H 4	0.22893	0.00000	0.76784	0.00323	0.77107	0.00020
C 5	-0.31241	1.99898	4.29228	0.02114	6.31241	0.09691
H 6	0.22853	0.00000	0.76810	0.00336	0.77147	0.00014
C 7	-0.21280	1.99879	4.19095	0.02306	6.21280	0.00295
C 8	-0.27897	1.99898	4.26030	0.01969	6.27897	0.09089
H 9	0.22641	0.00000	0.77028	0.00331	0.77359	0.00006
C 10	-0.35623	1.99899	4.33585	0.02139	6.35623	0.01911
H 11	0.22703	0.00000	0.76972	0.00325	0.77297	0.00015
C 12	-0.14253	1.99878	4.12185	0.02190	6.14253	0.07686
C 13	-0.38724	1.99899	4.36545	0.02280	6.38724	0.00272
H 14	0.22756	0.00000	0.76910	0.00333	0.77244	0.00009
C 15	-0.31320	1.99898	4.29364	0.02058	6.31320	0.06730
H 16	0.22685	0.00000	0.76988	0.00327	0.77315	0.00003
C 17	-0.17848	1.99878	4.15739	0.02231	6.17848	0.02482
C 18	-0.34941	1.99900	4.32781	0.02260	6.34941	0.12171
H 19	0.22998	0.00000	0.76656	0.00345	0.77002	0.00019
C 20	-0.35265	1.99900	4.33077	0.02289	6.35265	0.11664
H 21	0.22993	0.00000	0.76671	0.00336	0.77007	0.00017
C 22	-0.14703	1.99877	4.12694	0.02131	6.14703	0.03392
C 23	-0.37640	1.99898	4.35432	0.02310	6.37640	0.05215
H 24	0.22944	0.00000	0.76727	0.00330	0.77056	0.00008
C 25	-0.41714	1.99899	4.39390	0.02424	6.41714	0.00663
H 26	0.22987	0.00000	0.76673	0.00340	0.77013	0.00004

C	27	-0.13245	1.99877	4.11223	0.02144	6.13245	0.06488
C	28	-0.14343	1.99874	4.12353	0.02116	6.14343	0.05358
C	29	-0.17325	1.99873	4.15201	0.02251	6.17325	0.00216
C	30	-0.13338	1.99874	4.11367	0.02096	6.13338	0.05287
C	31	-0.16042	1.99873	4.13991	0.02177	6.16042	0.01987
C	32	-0.15830	1.99873	4.13819	0.02138	6.15830	0.02736
Cs	33	0.92618	53.98663	0.03178	0.05542	54.07382	0.01403
Cs	34	0.96110	53.99240	0.01211	0.03439	54.03890	0.00172
Cs	35	0.92063	53.98568	0.03767	0.05602	54.07937	0.01348
C	36	-0.36686	1.99900	4.34534	0.02253	6.36686	0.03605
H	37	0.22892	0.00000	0.76785	0.00323	0.77108	0.00020
C	38	-0.31242	1.99898	4.29230	0.02114	6.31242	0.09683
H	39	0.22853	0.00000	0.76810	0.00336	0.77147	0.00014
C	40	-0.21277	1.99879	4.19092	0.02306	6.21277	0.00293
C	41	-0.27892	1.99898	4.26025	0.01969	6.27892	0.09098
H	42	0.22641	0.00000	0.77028	0.00331	0.77359	0.00006
C	43	-0.35614	1.99899	4.33576	0.02138	6.35614	0.01927
H	44	0.22704	0.00000	0.76971	0.00325	0.77296	0.00015
C	45	-0.14256	1.99878	4.12188	0.02190	6.14256	0.07679
C	46	-0.38720	1.99899	4.36541	0.02280	6.38720	0.00274
H	47	0.22757	0.00000	0.76909	0.00333	0.77243	0.00009
C	48	-0.31337	1.99898	4.29381	0.02058	6.31337	0.06709
H	49	0.22685	0.00000	0.76988	0.00327	0.77315	0.00003
C	50	-0.17833	1.99878	4.15725	0.02230	6.17833	0.02495
C	51	-0.34957	1.99900	4.32796	0.02261	6.34957	0.12161
H	52	0.22999	0.00000	0.76655	0.00345	0.77001	0.00019
C	53	-0.35249	1.99900	4.33061	0.02288	6.35249	0.11686
H	54	0.22992	0.00000	0.76672	0.00336	0.77008	0.00017
C	55	-0.14720	1.99877	4.12711	0.02132	6.14720	0.03379
C	56	-0.37618	1.99898	4.35410	0.02310	6.37618	0.05238
H	57	0.22944	0.00000	0.76726	0.00330	0.77056	0.00008
C	58	-0.41729	1.99899	4.39404	0.02425	6.41729	0.00656
H	59	0.22987	0.00000	0.76673	0.00340	0.77013	0.00004
C	60	-0.13233	1.99877	4.11212	0.02144	6.13233	0.06499
C	61	-0.14335	1.99874	4.12346	0.02116	6.14335	0.05366
C	62	-0.17328	1.99873	4.15203	0.02251	6.17328	0.00214
C	63	-0.13343	1.99874	4.11372	0.02097	6.13343	0.05282
C	64	-0.16033	1.99873	4.13983	0.02177	6.16033	0.01998
C	65	-0.15840	1.99873	4.13828	0.02138	6.15840	0.02723
Cs	66	0.92617	53.98663	0.03179	0.05541	54.07383	0.01402
* Total *		0.00000	403.88436	184.87989	1.23575	590.00000	2.00000

Table S14. NBO charges for **1**$^{4-}$-*small* model (PBE0/def2-TZVP(Cs)//cc-pvDZ(C,H,O)).

Atom	No	Natural Charge	Natural Population			
			Core	Valence	Rydberg	Total
Cs	1	0.92541	53.97776	0.02874	0.06808	54.07459
Cs	2	0.92547	53.97779	0.02870	0.06805	54.07453
C	3	-0.17938	1.99889	4.15166	0.02883	6.17938
C	4	-0.17896	1.99889	4.15126	0.02881	6.17896
C	5	-0.17945	1.99889	4.15171	0.02885	6.17945
C	6	-0.17985	1.99889	4.15208	0.02888	6.17985
C	7	-0.17966	1.99889	4.15190	0.02887	6.17966
C	8	-0.18333	1.99899	4.15528	0.02905	6.18333
C	9	-0.38684	1.99919	4.36100	0.02665	6.38684
H	10	0.19085	0.00000	0.80644	0.00270	0.80915
C	11	-0.38738	1.99919	4.36153	0.02666	6.38738
H	12	0.19090	0.00000	0.80640	0.00270	0.80910
C	13	-0.18262	1.99899	4.15459	0.02903	6.18262
C	14	-0.38654	1.99919	4.36074	0.02662	6.38654
H	15	0.19084	0.00000	0.80646	0.00270	0.80916
C	16	-0.38677	1.99919	4.36096	0.02663	6.38677
H	17	0.19087	0.00000	0.80643	0.00270	0.80913
C	18	-0.18220	1.99899	4.15421	0.02900	6.18220
C	19	-0.38677	1.99919	4.36097	0.02662	6.38677
H	20	0.19086	0.00000	0.80644	0.00270	0.80914
C	21	-0.38648	1.99919	4.36068	0.02661	6.38648
H	22	0.19086	0.00000	0.80644	0.00270	0.80914
C	23	-0.18272	1.99899	4.15469	0.02904	6.18272
C	24	-0.38768	1.99919	4.36182	0.02668	6.38768
H	25	0.19091	0.00000	0.80639	0.00270	0.80909

C	26	-0.38712	1.99919	4.36126	0.02667	6.38712
H	27	0.19088	0.00000	0.80642	0.00270	0.80912
C	28	-0.18339	1.99899	4.15531	0.02908	6.18339
C	29	-0.38739	1.99919	4.36154	0.02666	6.38739
H	30	0.19091	0.00000	0.80639	0.00270	0.80909
C	31	-0.38708	1.99919	4.36125	0.02665	6.38708
H	32	0.19090	0.00000	0.80640	0.00270	0.80910
Cs	33	0.92543	53.97776	0.02875	0.06806	54.07457
C	34	-0.17969	1.99889	4.15193	0.02887	6.17969
C	35	-0.17944	1.99889	4.15170	0.02884	6.17944
C	36	-0.17918	1.99889	4.15144	0.02885	6.17918
C	37	-0.17925	1.99889	4.15152	0.02884	6.17925
C	38	-0.17959	1.99889	4.15185	0.02885	6.17959
C	39	-0.18267	1.99899	4.15465	0.02902	6.18267
C	40	-0.38721	1.99919	4.36136	0.02666	6.38721
H	41	0.19090	0.00000	0.80640	0.00270	0.80910
C	42	-0.38751	1.99919	4.36165	0.02668	6.38751
H	43	0.19090	0.00000	0.80640	0.00270	0.80910
C	44	-0.18294	1.99899	4.15489	0.02906	6.18294
C	45	-0.38774	1.99919	4.36185	0.02670	6.38774
H	46	0.19088	0.00000	0.80642	0.00270	0.80912
C	47	-0.38747	1.99919	4.36160	0.02668	6.38747
H	48	0.19088	0.00000	0.80642	0.00270	0.80912
C	49	-0.18289	1.99899	4.15486	0.02904	6.18289
C	50	-0.38724	1.99919	4.36140	0.02665	6.38724
H	51	0.19086	0.00000	0.80644	0.00270	0.80914
C	52	-0.38686	1.99919	4.36104	0.02663	6.38686
H	53	0.19089	0.00000	0.80641	0.00270	0.80911
C	54	-0.18272	1.99899	4.15470	0.02903	6.18272
C	55	-0.38652	1.99919	4.36072	0.02662	6.38652
H	56	0.19083	0.00000	0.80647	0.00270	0.80917
C	57	-0.38645	1.99919	4.36065	0.02662	6.38645
H	58	0.19083	0.00000	0.80647	0.00270	0.80917
C	59	-0.18258	1.99899	4.15457	0.02902	6.18258
C	60	-0.38663	1.99919	4.36084	0.02661	6.38663
H	61	0.19084	0.00000	0.80646	0.00270	0.80916
C	62	-0.38685	1.99919	4.36104	0.02663	6.38685
H	63	0.19090	0.00000	0.80640	0.00270	0.80910
Cs	64	0.95921	53.98882	0.01395	0.03803	54.04079
Cs	65	0.95919	53.98883	0.01392	0.03806	54.04081
Cs	66	0.92542	53.97770	0.02884	0.06803	54.07458
Cs	67	0.92542	53.97770	0.02886	0.06803	54.07458
* Total *		-1.00000	457.82893	186.58896	1.58212	646.00000

Table S15. NBO charges for **1**-*full* model (PBE0/def2-TZVP(Cs)//cc-pvDZ(C,H,O)).

			Natural Population				Natural
Atom	No	Natural Charge	Core	Valence	Rydberg	Total	Spin Density
Cs	1	0.91631	53.98815	0.04846	0.04708	54.08369	-0.00039
Cs	2	0.91631	53.98815	0.04846	0.04708	54.08369	-0.00039
Cs	3	0.90420	53.98003	0.05847	0.05729	54.09580	0.00365
Cs	4	0.90420	53.98003	0.05847	0.05729	54.09580	0.00365
O	5	-0.61726	1.99979	6.60658	0.01089	8.61726	-0.00004
O	6	-0.61726	1.99979	6.60658	0.01089	8.61726	-0.00004
O	7	-0.62234	1.99976	6.61091	0.01167	8.62234	0.00000
O	8	-0.62234	1.99976	6.61091	0.01167	8.62234	0.00000
O	9	-0.61801	1.99979	6.60739	0.01084	8.61801	-0.00004
O	10	-0.61801	1.99979	6.60738	0.01084	8.61801	-0.00004
O	11	-0.60815	1.99978	6.59735	0.01102	8.60815	-0.00002
O	12	-0.60816	1.99978	6.59736	0.01102	8.60816	-0.00002
O	13	-0.62064	1.99976	6.60846	0.01242	8.62064	0.00006
O	14	-0.62065	1.99976	6.60847	0.01242	8.62065	0.00006
O	15	-0.61749	1.99979	6.60677	0.01092	8.61749	0.00000
O	16	-0.61751	1.99979	6.60679	0.01092	8.61751	0.00000
C	17	-0.37628	1.99899	4.35522	0.02207	6.37628	0.01695
C	18	-0.37628	1.99899	4.35522	0.02207	6.37628	0.01701
H	19	0.21410	0.00000	0.78250	0.00340	0.78590	-0.00057
H	20	0.21411	0.00000	0.78249	0.00340	0.78589	-0.00057
C	21	-0.39060	1.99899	4.36992	0.02170	6.39060	-0.00579
C	22	-0.39062	1.99899	4.36993	0.02170	6.39062	-0.00584
H	23	0.21485	0.00000	0.78188	0.00327	0.78515	0.00010

H 24	0.21487	0.00000	0.78187	0.00327	0.78513	0.00011
C 25	-0.12660	1.99876	4.10733	0.02051	6.12660	0.06960
C 26	-0.12658	1.99876	4.10732	0.02051	6.12658	0.06962
C 27	-0.34275	1.99900	4.32379	0.01997	6.34275	0.03626
C 28	-0.34276	1.99900	4.32379	0.01997	6.34276	0.03626
H 29	0.21516	0.00000	0.78141	0.00343	0.78484	-0.00099
H 30	0.21516	0.00000	0.78141	0.00343	0.78484	-0.00099
C 31	-0.26960	1.99898	4.25102	0.01959	6.26960	0.15336
C 32	-0.26956	1.99898	4.25099	0.01959	6.26956	0.15330
H 33	0.21890	0.00000	0.77767	0.00343	0.78110	-0.00473
H 34	0.21889	0.00000	0.77768	0.00343	0.78111	-0.00473
C 35	-0.16667	1.99876	4.14654	0.02137	6.16667	-0.03045
C 36	-0.16669	1.99876	4.14657	0.02137	6.16669	-0.03043
C 37	-0.32665	1.99898	4.30651	0.02115	6.32665	0.13169
C 38	-0.32662	1.99898	4.30649	0.02115	6.32662	0.13169
H 39	0.21986	0.00000	0.77691	0.00324	0.78014	-0.00413
H 40	0.21985	0.00000	0.77691	0.00324	0.78015	-0.00413
C 41	-0.41904	1.99901	4.39743	0.02260	6.41904	-0.02881
C 42	-0.41903	1.99901	4.39742	0.02260	6.41903	-0.02881
H 43	0.21369	0.00000	0.78314	0.00317	0.78631	0.00095
H 44	0.21369	0.00000	0.78314	0.00317	0.78631	0.00095
C 45	-0.10875	1.99877	4.08979	0.02019	6.10875	0.10544
C 46	-0.10876	1.99877	4.08981	0.02019	6.10876	0.10546
C 47	-0.43002	1.99900	4.40814	0.02288	6.43002	-0.03079
C 48	-0.43004	1.99900	4.40817	0.02288	6.43004	-0.03076
H 49	0.21972	0.00000	0.77708	0.00320	0.78028	0.00081
H 50	0.21972	0.00000	0.77708	0.00320	0.78028	0.00081
C 51	-0.34138	1.99898	4.32080	0.02160	6.34138	0.15222
C 52	-0.34135	1.99898	4.32077	0.02160	6.34135	0.15221
H 53	0.22318	0.00000	0.77356	0.00326	0.77682	-0.00472
H 54	0.22318	0.00000	0.77356	0.00326	0.77682	-0.00472
C 55	-0.16665	1.99876	4.14613	0.02177	6.16665	-0.04190
C 56	-0.16667	1.99876	4.14614	0.02177	6.16667	-0.04189
C 57	-0.30809	1.99898	4.28792	0.02119	6.30809	0.19275
C 58	-0.30807	1.99898	4.28790	0.02119	6.30807	0.19271
H 59	0.21461	0.00000	0.78166	0.00373	0.78539	-0.00586
H 60	0.21461	0.00000	0.78165	0.00373	0.78539	-0.00586
C 61	-0.38801	1.99900	4.36644	0.02257	6.38801	0.07694
C 62	-0.38799	1.99900	4.36643	0.02257	6.38799	0.07686
H 63	0.21316	0.00000	0.78338	0.00346	0.78684	-0.00229
H 64	0.21316	0.00000	0.78338	0.00346	0.78684	-0.00229
C 65	-0.13203	1.99876	4.11245	0.02082	6.13203	0.04827
C 66	-0.13203	1.99876	4.11246	0.02082	6.13203	0.04820
C 67	-0.13333	1.99873	4.11422	0.02037	6.13333	0.04634
C 68	-0.13333	1.99873	4.11423	0.02037	6.13333	0.04633
C 69	-0.12867	1.99874	4.10997	0.01996	6.12867	0.03399
C 70	-0.12867	1.99874	4.10997	0.01996	6.12867	0.03400
C 71	-0.15321	1.99873	4.13415	0.02033	6.15321	-0.00367
C 72	-0.15320	1.99873	4.13414	0.02033	6.15320	-0.00368
C 73	-0.12566	1.99874	4.10699	0.01993	6.12566	0.08056
C 74	-0.12567	1.99874	4.10700	0.01993	6.12567	0.08055
C 75	-0.14529	1.99871	4.12485	0.02173	6.14529	0.00099
C 76	-0.14530	1.99871	4.12486	0.02173	6.14530	0.00098
C 77	-0.27482	1.99935	4.25999	0.01547	6.27482	0.00033
C 78	-0.27482	1.99935	4.25999	0.01547	6.27482	0.00033
H 79	0.20751	0.00000	0.78824	0.00425	0.79249	0.00007
H 80	0.20751	0.00000	0.78824	0.00425	0.79249	0.00006
H 81	0.20704	0.00000	0.79057	0.00239	0.79296	0.00001
H 82	0.20704	0.00000	0.79057	0.00239	0.79296	0.00001
H 83	0.17660	0.00000	0.81941	0.00399	0.82340	-0.00003
H 84	0.17660	0.00000	0.81941	0.00399	0.82340	-0.00003
C 85	-0.09655	1.99916	4.07768	0.01971	6.09655	0.00017
C 86	-0.09655	1.99916	4.07768	0.01970	6.09655	0.00017
H 87	0.22044	0.00000	0.77352	0.00604	0.77956	0.00033
H 88	0.22044	0.00000	0.77352	0.00604	0.77956	0.00033
H 89	0.19524	0.00000	0.80015	0.00461	0.80476	-0.00002
H 90	0.19524	0.00000	0.80015	0.00461	0.80476	-0.00002
C 91	-0.08482	1.99917	4.06609	0.01956	6.08482	0.00000
C 92	-0.08482	1.99917	4.06610	0.01956	6.08482	0.00000
H 93	0.19483	0.00000	0.79873	0.00644	0.80517	0.00000
H 94	0.19484	0.00000	0.79872	0.00644	0.80516	0.00000
H 95	0.19900	0.00000	0.79624	0.00476	0.80100	-0.00002
H 96	0.19901	0.00000	0.79623	0.00476	0.80099	-0.00002
C 97	-0.08488	1.99917	4.06617	0.01954	6.08488	0.00001
C 98	-0.08488	1.99917	4.06617	0.01954	6.08488	0.00001
H 99	0.19828	0.00000	0.79698	0.00474	0.80172	0.00001
H100	0.19828	0.00000	0.79698	0.00474	0.80172	0.00001

H101	0.19509	0.00000	0.79856	0.00636	0.80491	0.00005
H102	0.19509	0.00000	0.79856	0.00636	0.80491	0.00005
C103	-0.09670	1.99916	4.07797	0.01957	6.09670	0.00026
C104	-0.09670	1.99916	4.07797	0.01957	6.09670	0.00026
H105	0.19461	0.00000	0.80073	0.00466	0.80539	-0.00002
H106	0.19461	0.00000	0.80073	0.00466	0.80539	-0.00002
H107	0.22163	0.00000	0.77224	0.00613	0.77837	0.00005
H108	0.22163	0.00000	0.77224	0.00613	0.77837	0.00005
C109	-0.27407	1.99935	4.25924	0.01548	6.27407	0.00022
C110	-0.27407	1.99935	4.25924	0.01548	6.27407	0.00022
H111	0.17663	0.00000	0.81939	0.00398	0.82337	0.00000
H112	0.17663	0.00000	0.81939	0.00398	0.82337	0.00000
H113	0.20935	0.00000	0.78821	0.00244	0.79065	-0.00004
H114	0.20935	0.00000	0.78821	0.00244	0.79065	-0.00004
H115	0.20525	0.00000	0.79028	0.00447	0.79475	0.00031
H116	0.20525	0.00000	0.79028	0.00447	0.79475	0.00031
C117	-0.27134	1.99935	4.25584	0.01616	6.27134	0.00090
C118	-0.27134	1.99935	4.25583	0.01616	6.27134	0.00090
H119	0.17833	0.00000	0.81774	0.00393	0.82167	0.00017
H120	0.17833	0.00000	0.81774	0.00393	0.82167	0.00017
H121	0.20514	0.00000	0.79210	0.00276	0.79486	0.00000
H122	0.20514	0.00000	0.79211	0.00276	0.79486	0.00000
H123	0.20168	0.00000	0.79335	0.00497	0.79832	0.00115
H124	0.20168	0.00000	0.79335	0.00497	0.79832	0.00115
C125	-0.09277	1.99916	4.07368	0.01992	6.09277	0.00005
C126	-0.09277	1.99916	4.07368	0.01992	6.09277	0.00005
H127	0.19437	0.00000	0.80099	0.00464	0.80563	-0.00001
H128	0.19437	0.00000	0.80099	0.00464	0.80563	-0.00001
H129	0.20932	0.00000	0.78356	0.00711	0.79068	0.00008
H130	0.20932	0.00000	0.78356	0.00711	0.79068	0.00008
C131	-0.08469	1.99917	4.06579	0.01973	6.08469	0.00007
C132	-0.08468	1.99917	4.06579	0.01973	6.08468	0.00007
H133	0.19583	0.00000	0.79964	0.00453	0.80417	0.00001
H134	0.19582	0.00000	0.79964	0.00453	0.80418	0.00001
H135	0.19940	0.00000	0.79441	0.00619	0.80060	0.00000
H136	0.19940	0.00000	0.79441	0.00619	0.80060	0.00000
C137	-0.10104	1.99919	4.08419	0.01766	6.10104	0.00004
C138	-0.10104	1.99919	4.08419	0.01766	6.10104	0.00004
H139	0.20463	0.00000	0.79056	0.00481	0.79537	0.00007
H140	0.20463	0.00000	0.79056	0.00481	0.79537	0.00007
H141	0.18544	0.00000	0.80953	0.00502	0.81456	0.00015
H142	0.18545	0.00000	0.80953	0.00502	0.81455	0.00015
C143	-0.09313	1.99913	4.06955	0.02445	6.09313	0.00172
C144	-0.09313	1.99913	4.06955	0.02444	6.09313	0.00172
H145	0.19385	0.00000	0.80142	0.00473	0.80615	-0.00002
H146	0.19384	0.00000	0.80143	0.00473	0.80616	-0.00002
H147	0.23877	0.00000	0.75420	0.00704	0.76123	0.00127
H148	0.23877	0.00000	0.75419	0.00704	0.76123	0.00127
C149	-0.27174	1.99935	4.25452	0.01787	6.27174	0.00034
C150	-0.27176	1.99935	4.25453	0.01788	6.27176	0.00034
H151	0.18682	0.00000	0.80833	0.00485	0.81318	0.00006
H152	0.18682	0.00000	0.80834	0.00485	0.81318	0.00006
H153	0.21517	0.00000	0.78089	0.00394	0.78483	0.00107
H154	0.21518	0.00000	0.78089	0.00394	0.78482	0.00107
H155	0.17929	0.00000	0.81575	0.00496	0.82071	-0.00002
H156	0.17929	0.00000	0.81575	0.00496	0.82071	-0.00002
Cs157	0.89709	53.97911	0.06376	0.06003	54.10291	0.00393
Cs158	0.89709	53.97911	0.06376	0.06003	54.10291	0.00393
O159	-0.61448	1.99978	6.60349	0.01121	8.61448	0.00002
O160	-0.61447	1.99978	6.60348	0.01121	8.61447	0.00002
O161	-0.61034	1.99976	6.59823	0.01235	8.61034	0.00000
O162	-0.61034	1.99976	6.59823	0.01235	8.61034	0.00000
O163	-0.61479	1.99978	6.60385	0.01116	8.61479	0.00032
O164	-0.61479	1.99978	6.60385	0.01116	8.61479	0.00032
C165	-0.27018	1.99936	4.25498	0.01585	6.27018	0.00005
C166	-0.27019	1.99936	4.25498	0.01585	6.27019	0.00005
H167	0.18814	0.00000	0.80803	0.00383	0.81186	0.00003
H168	0.18814	0.00000	0.80803	0.00383	0.81186	0.00003
H169	0.22297	0.00000	0.77219	0.00484	0.77703	0.00007
H170	0.22297	0.00000	0.77218	0.00484	0.77703	0.00007
H171	0.17588	0.00000	0.82013	0.00399	0.82412	0.00005
H172	0.17588	0.00000	0.82013	0.00399	0.82412	0.00005
C173	-0.08541	1.99918	4.06702	0.01921	6.08541	0.00002
C174	-0.08542	1.99918	4.06702	0.01921	6.08542	0.00002
H175	0.20061	0.00000	0.79328	0.00611	0.79939	0.00001
H176	0.20061	0.00000	0.79328	0.00611	0.79939	0.00001
H177	0.19461	0.00000	0.80072	0.00466	0.80539	0.00000

H178	0.19461	0.00000	0.80073	0.00466	0.80539	0.00000
C179	-0.08684	1.99918	4.06773	0.01993	6.08684	0.00016
C180	-0.08684	1.99918	4.06773	0.01993	6.08684	0.00016
H181	0.19608	0.00000	0.79784	0.00608	0.80392	0.00040
H182	0.19608	0.00000	0.79784	0.00608	0.80392	0.00040
H183	0.19729	0.00000	0.79808	0.00462	0.80271	0.00001
H184	0.19729	0.00000	0.79808	0.00462	0.80271	0.00001
C185	-0.08967	1.99916	4.07050	0.02001	6.08967	-0.00007
C186	-0.08966	1.99916	4.07050	0.02001	6.08966	-0.00007
H187	0.19633	0.00000	0.79920	0.00448	0.80367	-0.00001
H188	0.19633	0.00000	0.79920	0.00448	0.80367	-0.00001
H189	0.20389	0.00000	0.78968	0.00643	0.79611	-0.00007
H190	0.20389	0.00000	0.78968	0.00643	0.79611	-0.00007
C191	-0.08334	1.99918	4.06482	0.01934	6.08334	0.00020
C192	-0.08333	1.99918	4.06481	0.01934	6.08333	0.00020
H193	0.20050	0.00000	0.79487	0.00463	0.79950	0.00004
H194	0.20050	0.00000	0.79487	0.00463	0.79950	0.00004
H195	0.19512	0.00000	0.79892	0.00597	0.80488	-0.00001
H196	0.19512	0.00000	0.79892	0.00597	0.80488	-0.00001
C197	-0.27425	1.99936	4.25847	0.01642	6.27425	0.00038
C198	-0.27426	1.99936	4.25847	0.01642	6.27426	0.00038
H199	0.17878	0.00000	0.81709	0.00414	0.82122	0.00005
H200	0.17878	0.00000	0.81709	0.00414	0.82122	0.00005
H201	0.23165	0.00000	0.76439	0.00397	0.76835	-0.00006
H202	0.23165	0.00000	0.76439	0.00397	0.76835	-0.00006
H203	0.18279	0.00000	0.81323	0.00398	0.81721	0.00001
H204	0.18280	0.00000	0.81322	0.00398	0.81720	0.00001
* Total *	0.00000	511.81754	519.66021	2.52224	*********	2.00000

Table S16. NBO charges for **1H**-*full* model (PBE0/def2-TZVP(Cs)//cc-pvDZ(C,H,O)).

		Natural Population				
Atom No	Natural Charge	Core	Valence	Rydberg	Total	Natural Spin Density
Cs 1	0.91673	53.98673	0.05097	0.04557	54.08327	-0.00057
Cs 2	0.89976	53.97487	0.06803	0.05733	54.10024	0.00380
O 3	-0.61493	1.99980	6.60454	0.01059	8.61493	0.00003
O 4	-0.62049	1.99977	6.60918	0.01154	8.62049	-0.00002
O 5	-0.61135	1.99979	6.60085	0.01071	8.61135	-0.00002
O 6	-0.61285	1.99980	6.60263	0.01042	8.61285	0.00004
O 7	-0.62875	1.99977	6.61679	0.01220	8.62875	0.00005
O 8	-0.61955	1.99981	6.60936	0.01039	8.61955	0.00005
C 9	-0.39737	1.99898	4.37463	0.02376	6.39737	-0.02454
H 10	0.21901	0.00000	0.77775	0.00324	0.78099	0.00101
C 11	-0.32532	1.99897	4.30564	0.02071	6.32532	0.14582
H 12	0.21693	0.00000	0.77978	0.00328	0.78307	-0.00458
C 13	-0.17561	1.99878	4.15591	0.02091	6.17561	-0.03641
C 14	-0.29052	1.99898	4.27230	0.01924	6.29052	0.14547
H 15	0.21316	0.00000	0.78329	0.00354	0.78684	-0.00460
C 16	-0.31855	1.99897	4.29859	0.02099	6.31855	0.02273
H 17	0.22053	0.00000	0.77616	0.00330	0.77947	-0.00066
C 18	-0.10543	1.99875	4.08639	0.02030	6.10543	0.08583
C 19	-0.38741	1.99899	4.36608	0.02235	6.38741	-0.02357
H 20	0.22106	0.00000	0.77581	0.00312	0.77894	0.00065
C 21	-0.37375	1.99897	4.35396	0.02082	6.37375	0.03837
H 22	0.21326	0.00000	0.78344	0.00331	0.78674	-0.00139
C 23	-0.15205	1.99876	4.13212	0.02117	6.15205	0.02855
C 24	-0.38809	1.99898	4.36654	0.02258	6.38809	0.10162
H 25	0.22130	0.00000	0.77537	0.00332	0.77870	-0.00310
C 26	-0.33206	1.99897	4.31063	0.02246	6.33206	0.18704
H 27	0.22519	0.00000	0.77163	0.00318	0.77481	-0.00558
C 28	-0.16340	1.99872	4.14226	0.02242	6.16340	-0.03396
C 29	-0.34350	1.99897	4.32250	0.02203	6.34350	0.13369
H 30	0.21537	0.00000	0.78120	0.00343	0.78463	-0.00420
C 31	-0.42904	1.99897	4.40665	0.02342	6.42904	-0.03514
H 32	0.21990	0.00000	0.77666	0.00344	0.78010	0.00093
C 33	-0.10848	1.99876	4.08894	0.02079	6.10848	0.11325
C 34	-0.12623	1.99871	4.10757	0.01995	6.12623	0.07066
C 35	-0.15098	1.99873	4.13130	0.02094	6.15098	-0.00377
C 36	-0.12712	1.99870	4.10893	0.01949	6.12712	0.03113
C 37	-0.14669	1.99875	4.12677	0.02117	6.14669	0.04804
C 38	-0.14353	1.99872	4.12317	0.02164	6.14353	0.00931

C 39	-0.28431	1.99935	4.26988	0.01508	6.28431	-0.00010
H 40	0.20694	0.00000	0.78896	0.00410	0.79306	0.00019
H 41	0.20657	0.00000	0.79109	0.00234	0.79343	-0.00001
H 42	0.18212	0.00000	0.81390	0.00398	0.81788	0.00001
C 43	-0.10017	1.99911	4.08097	0.02009	6.10017	0.00000
H 44	0.22275	0.00000	0.77046	0.00679	0.77725	0.00007
H 45	0.19501	0.00000	0.80046	0.00453	0.80499	0.00000
C 46	-0.08830	1.99915	4.06979	0.01936	6.08830	-0.00001
H 47	0.19371	0.00000	0.80003	0.00626	0.80629	0.00014
H 48	0.20451	0.00000	0.79073	0.00477	0.79549	-0.00004
C 49	-0.08857	1.99917	4.07048	0.01892	6.08857	0.00005
H 50	0.20579	0.00000	0.78942	0.00479	0.79421	0.00005
H 51	0.19394	0.00000	0.79999	0.00607	0.80606	0.00007
C 52	-0.10282	1.99913	4.08429	0.01939	6.10282	-0.00015
H 53	0.19594	0.00000	0.79958	0.00448	0.80406	0.00000
H 54	0.22293	0.00000	0.77091	0.00616	0.77707	0.00005
C 55	-0.28099	1.99938	4.26648	0.01513	6.28099	-0.00004
H 56	0.18306	0.00000	0.81290	0.00404	0.81694	0.00001
H 57	0.20900	0.00000	0.78868	0.00231	0.79100	0.00000
H 58	0.19535	0.00000	0.80004	0.00461	0.80465	-0.00003
C 59	-0.27126	1.99933	4.25581	0.01613	6.27126	0.00020
H 60	0.17947	0.00000	0.81661	0.00392	0.82053	0.00009
H 61	0.20969	0.00000	0.78732	0.00299	0.79031	0.00004
H 62	0.20081	0.00000	0.79401	0.00518	0.79919	0.00002
C 63	-0.10115	1.99913	4.08233	0.01970	6.10115	0.00042
H 64	0.20115	0.00000	0.79424	0.00462	0.79885	0.00002
H 65	0.21310	0.00000	0.78045	0.00646	0.78690	0.00048
C 66	-0.09191	1.99912	4.07315	0.01964	6.09191	-0.00001
H 67	0.20280	0.00000	0.79283	0.00437	0.79720	0.00002
H 68	0.20675	0.00000	0.78735	0.00590	0.79325	0.00006
C 69	-0.10448	1.99918	4.08802	0.01728	6.10448	0.00001
H 70	0.20683	0.00000	0.78854	0.00463	0.79317	0.00009
H 71	0.19032	0.00000	0.80479	0.00489	0.80968	-0.00001
C 72	-0.09576	1.99913	4.07284	0.02379	6.09576	0.00099
H 73	0.19838	0.00000	0.79683	0.00479	0.80162	0.00007
H 74	0.23579	0.00000	0.75715	0.00706	0.76421	0.00129
C 75	-0.27965	1.99934	4.26263	0.01768	6.27965	0.00101
H 76	0.19136	0.00000	0.80374	0.00490	0.80864	-0.00004
H 77	0.22296	0.00000	0.77218	0.00486	0.77704	0.00106
H 78	0.17937	0.00000	0.81649	0.00414	0.82063	0.00006
Cs 79	0.89300	53.97416	0.07509	0.05774	54.10700	0.00342
Cs 80	0.89300	53.97416	0.07509	0.05774	54.10700	0.00342
Cs 81	0.89977	53.97487	0.06803	0.05733	54.10023	0.00380
C 82	-0.39730	1.99898	4.37456	0.02376	6.39730	-0.02459
C 83	-0.32532	1.99897	4.30564	0.02072	6.32532	0.14582
C 84	-0.17551	1.99878	4.15581	0.02092	6.17551	-0.03642
C 85	-0.12624	1.99871	4.10757	0.01995	6.12624	0.07064
C 86	-0.15095	1.99873	4.13127	0.02094	6.15095	-0.00376
O 87	-0.61285	1.99979	6.60234	0.01073	8.61285	0.00001
O 88	-0.60775	1.99977	6.59599	0.01199	8.60775	0.00000
O 89	-0.60891	1.99979	6.59829	0.01083	8.60891	0.00028
C 90	-0.27457	1.99934	4.25953	0.01570	6.27457	0.00063
H 91	0.18906	0.00000	0.80717	0.00376	0.81094	-0.00003
H 92	0.22858	0.00000	0.76683	0.00459	0.77142	0.00047
H 93	0.17263	0.00000	0.82334	0.00404	0.82737	0.00000
C 94	-0.09034	1.99915	4.07218	0.01901	6.09034	0.00001
H 95	0.20012	0.00000	0.79381	0.00608	0.79988	0.00001
H 96	0.19635	0.00000	0.79914	0.00451	0.80365	0.00001
C 97	-0.09488	1.99915	4.07575	0.01999	6.09488	0.00013
H 98	0.19960	0.00000	0.79308	0.00732	0.80040	0.00019
H 99	0.19929	0.00000	0.79614	0.00457	0.80071	0.00001
C100	-0.09479	1.99914	4.07626	0.01939	6.09479	0.00064
H101	0.19628	0.00000	0.79929	0.00442	0.80372	0.00005
H102	0.21192	0.00000	0.78210	0.00598	0.78808	0.00021
C103	-0.08890	1.99917	4.07078	0.01895	6.08890	0.00020
H104	0.20198	0.00000	0.79344	0.00458	0.79802	0.00000
H105	0.19584	0.00000	0.79837	0.00579	0.80416	0.00004
C106	-0.28576	1.99935	4.26977	0.01664	6.28576	0.00096
H107	0.17885	0.00000	0.81687	0.00428	0.82115	0.00032
H108	0.23460	0.00000	0.76104	0.00436	0.76540	0.00024
H109	0.18455	0.00000	0.81169	0.00376	0.81545	0.00011
C110	-0.16343	1.99872	4.14229	0.02242	6.16343	-0.03398
C111	-0.34344	1.99897	4.32244	0.02203	6.34344	0.13372
C112	-0.42915	1.99897	4.40676	0.02342	6.42915	-0.03513
C113	-0.10848	1.99876	4.08893	0.02079	6.10848	0.11324
C114	-0.14356	1.99872	4.12320	0.02164	6.14356	0.00931
Cs115	0.91674	53.98672	0.05098	0.04555	54.08326	-0.00058

O116	-0.61493	1.99980	6.60455	0.01059	8.61493	0.00003
O117	-0.62048	1.99977	6.60916	0.01154	8.62048	-0.00002
O118	-0.61133	1.99979	6.60083	0.01070	8.61133	-0.00002
O119	-0.61287	1.99980	6.60265	0.01042	8.61287	0.00004
O120	-0.62876	1.99977	6.61680	0.01220	8.62876	0.00005
O121	-0.61954	1.99981	6.60935	0.01039	8.61954	0.00005
H122	0.21900	0.00000	0.77777	0.00324	0.78100	0.00101
H123	0.21689	0.00000	0.77982	0.00328	0.78311	-0.00458
C124	-0.29053	1.99898	4.27230	0.01925	6.29053	0.14553
H125	0.21317	0.00000	0.78328	0.00354	0.78683	-0.00460
C126	-0.31863	1.99897	4.29867	0.02098	6.31863	0.02280
H127	0.22054	0.00000	0.77616	0.00330	0.77946	-0.00067
C128	-0.10543	1.99875	4.08638	0.02029	6.10543	0.08578
C129	-0.38743	1.99899	4.36609	0.02235	6.38743	-0.02351
H130	0.22106	0.00000	0.77582	0.00312	0.77894	0.00064
C131	-0.37373	1.99897	4.35395	0.02082	6.37373	0.03830
H132	0.21327	0.00000	0.78342	0.00331	0.78673	-0.00138
C133	-0.15203	1.99876	4.13211	0.02117	6.15203	0.02859
C134	-0.38817	1.99898	4.36661	0.02258	6.38817	0.10155
H135	0.22130	0.00000	0.77537	0.00332	0.77870	-0.00309
C136	-0.33201	1.99897	4.31058	0.02246	6.33201	0.18707
H137	0.22519	0.00000	0.77163	0.00318	0.77481	-0.00558
H138	0.21536	0.00000	0.78120	0.00343	0.78464	-0.00420
H139	0.21992	0.00000	0.77664	0.00344	0.78008	0.00093
C140	-0.12714	1.99870	4.10894	0.01949	6.12714	0.03111
C141	-0.14668	1.99875	4.12676	0.02117	6.14668	0.04805
C142	-0.28421	1.99935	4.26978	0.01508	6.28421	-0.00010
H143	0.20681	0.00000	0.78909	0.00410	0.79319	0.00020
H144	0.20664	0.00000	0.79102	0.00234	0.79336	-0.00001
H145	0.18210	0.00000	0.81391	0.00399	0.81790	0.00001
C146	-0.10018	1.99911	4.08099	0.02009	6.10018	0.00000
H147	0.22274	0.00000	0.77047	0.00679	0.77726	0.00007
H148	0.19504	0.00000	0.80043	0.00453	0.80496	0.00000
C149	-0.08830	1.99915	4.06979	0.01936	6.08830	-0.00001
H150	0.19371	0.00000	0.80003	0.00626	0.80629	0.00014
H151	0.20451	0.00000	0.79072	0.00477	0.79549	-0.00004
C152	-0.08857	1.99917	4.07049	0.01891	6.08857	0.00005
H153	0.20580	0.00000	0.78942	0.00479	0.79420	0.00005
H154	0.19393	0.00000	0.79999	0.00608	0.80607	0.00007
C155	-0.10281	1.99913	4.08429	0.01939	6.10281	-0.00015
H156	0.19595	0.00000	0.79957	0.00448	0.80405	0.00000
H157	0.22292	0.00000	0.77092	0.00616	0.77708	0.00005
C158	-0.28099	1.99938	4.26648	0.01513	6.28099	-0.00004
H159	0.18303	0.00000	0.81293	0.00404	0.81697	0.00001
H160	0.20907	0.00000	0.78862	0.00231	0.79093	0.00000
H161	0.19529	0.00000	0.80010	0.00461	0.80471	-0.00003
C162	-0.27140	1.99933	4.25594	0.01613	6.27140	0.00020
H163	0.17947	0.00000	0.81661	0.00392	0.82053	0.00009
H164	0.20965	0.00000	0.78737	0.00298	0.79035	0.00003
H165	0.20096	0.00000	0.79385	0.00520	0.79904	0.00002
C166	-0.10113	1.99913	4.08231	0.01970	6.10113	0.00042
H167	0.20116	0.00000	0.79423	0.00461	0.79884	0.00002
H168	0.21308	0.00000	0.78046	0.00646	0.78692	0.00048
C169	-0.09191	1.99912	4.07315	0.01964	6.09191	-0.00001
H170	0.20280	0.00000	0.79283	0.00437	0.79720	0.00002
H171	0.20673	0.00000	0.78736	0.00590	0.79327	0.00006
C172	-0.10448	1.99918	4.08802	0.01728	6.10448	0.00001
H173	0.20683	0.00000	0.78854	0.00463	0.79317	0.00009
H174	0.19031	0.00000	0.80480	0.00489	0.80969	-0.00001
C175	-0.09574	1.99913	4.07283	0.02379	6.09574	0.00099
H176	0.19837	0.00000	0.79684	0.00479	0.80163	0.00007
H177	0.23580	0.00000	0.75714	0.00706	0.76420	0.00129
C178	-0.27964	1.99934	4.26261	0.01768	6.27964	0.00101
H179	0.19136	0.00000	0.80374	0.00490	0.80864	-0.00004
H180	0.22295	0.00000	0.77219	0.00486	0.77705	0.00106
H181	0.17936	0.00000	0.81649	0.00414	0.82064	0.00006
O182	-0.61286	1.99979	6.60235	0.01073	8.61286	0.00001
O183	-0.60776	1.99977	6.59600	0.01199	8.60776	0.00000
O184	-0.60891	1.99979	6.59828	0.01084	8.60891	0.00028
C185	-0.27458	1.99934	4.25954	0.01570	6.27458	0.00063
H186	0.18906	0.00000	0.80718	0.00376	0.81094	-0.00003
H187	0.22860	0.00000	0.76681	0.00459	0.77140	0.00047
H188	0.17263	0.00000	0.82333	0.00404	0.82737	0.00000
C189	-0.09034	1.99915	4.07218	0.01901	6.09034	0.00001
H190	0.20011	0.00000	0.79381	0.00608	0.79989	0.00001
H191	0.19635	0.00000	0.79914	0.00451	0.80365	0.00001
C192	-0.09488	1.99915	4.07574	0.01999	6.09488	0.00013

H193	0.19959	0.00000	0.79310	0.00731	0.80041	0.00019
H194	0.19929	0.00000	0.79614	0.00457	0.80071	0.00001
C195	-0.09479	1.99914	4.07625	0.01939	6.09479	0.00064
H196	0.19628	0.00000	0.79930	0.00442	0.80372	0.00005
H197	0.21191	0.00000	0.78210	0.00598	0.78809	0.00021
C198	-0.08890	1.99917	4.07078	0.01895	6.08890	0.00020
H199	0.20198	0.00000	0.79344	0.00458	0.79802	0.00000
H200	0.19584	0.00000	0.79837	0.00579	0.80416	0.00004
C201	-0.28593	1.99935	4.26995	0.01663	6.28593	0.00096
H202	0.17885	0.00000	0.81687	0.00428	0.82115	0.00032
H203	0.23481	0.00000	0.76084	0.00435	0.76519	0.00024
H204	0.18447	0.00000	0.81178	0.00376	0.81553	0.00011
* Total *	0.00000	511.79357	519.69800	2.50843	*********	2.00000

Table S17. NBO charges for "naked" $C_{20}H_{10}^{\bullet 3-}$ (PBE0/cc-pvDZ).

Atom No	Natural Charge	Natural Population: Core	Valence	Rydberg	Total	Natural Spin Density
C 1	-0.32044	1.99902	4.30368	0.01774	6.32044	0.17076
C 2	-0.38095	1.99902	4.36365	0.01827	6.38095	0.01351
C 3	-0.36522	1.99904	4.34758	0.01860	6.36522	0.09059
C 4	-0.32889	1.99901	4.31233	0.01755	6.32889	0.12764
C 5	-0.39981	1.99904	4.38194	0.01882	6.39981	-0.03376
H 6	0.15712	0.00000	0.83801	0.00487	0.84288	-0.00546
H 7	0.15497	0.00000	0.84034	0.00469	0.84503	-0.00057
H 8	0.15760	0.00000	0.83767	0.00473	0.84240	-0.00294
H 9	0.15570	0.00000	0.83945	0.00486	0.84430	-0.00413
H 10	0.15619	0.00000	0.83917	0.00464	0.84381	0.00090
C 11	-0.09902	1.99879	4.08199	0.01823	6.09902	0.02270
C 12	-0.09352	1.99879	4.07655	0.01818	6.09352	0.03692
C 13	-0.13115	1.99880	4.11376	0.01860	6.13115	-0.04444
C 14	-0.08229	1.99879	4.06540	0.01810	6.08229	0.07544
C 15	-0.12627	1.99880	4.10893	0.01854	6.12627	-0.03519
C 16	-0.08227	1.99878	4.06487	0.01862	6.08227	0.05644
C 17	-0.07440	1.99879	4.05703	0.01858	6.07440	0.07100
C 18	-0.11739	1.99877	4.09981	0.01881	6.11739	-0.00708
C 19	-0.05284	1.99880	4.03556	0.01849	6.05284	0.11087
C 20	-0.11325	1.99877	4.09569	0.01879	6.11325	0.00038
C 21	-0.35546	1.99903	4.33796	0.01846	6.35546	0.11473
C 22	-0.38907	1.99903	4.37163	0.01842	6.38907	-0.00554
C 23	-0.31659	1.99902	4.29996	0.01761	6.31659	0.17278
C 24	-0.39663	1.99904	4.37874	0.01885	6.39663	-0.02047
C 25	-0.33770	1.99901	4.32103	0.01765	6.33770	0.10712
H 26	0.15762	0.00000	0.83762	0.00476	0.84238	-0.00369
H 27	0.15506	0.00000	0.84027	0.00467	0.84494	0.00002
H 28	0.15688	0.00000	0.83824	0.00489	0.84312	-0.00553
H 29	0.15657	0.00000	0.83878	0.00465	0.84343	0.00050
H 30	0.15546	0.00000	0.83971	0.00483	0.84454	-0.00349
* Total *	-3.00000	39.97814	92.60736	0.41450	133.00000	1.00000

Table S18. NBO charges for neutral $C_{20}H_{10}$ (PBE0/cc-pvDZ).

Atom No	Natural Charge	Natural Population: Core	Valence	Rydberg	Total
C 1	-0.20947	1.99897	4.19404	0.01646	6.20947
C 2	-0.20682	1.99898	4.19155	0.01628	6.20682
C 3	-0.20223	1.99899	4.18686	0.01638	6.20223
C 4	-0.21196	1.99897	4.19660	0.01638	6.21196
C 5	-0.20067	1.99900	4.18541	0.01626	6.20067
H 6	0.23997	0.00000	0.75680	0.00323	0.76003
H 7	0.24035	0.00000	0.75651	0.00314	0.75965
H 8	0.23958	0.00000	0.75724	0.00318	0.76042
H 9	0.24042	0.00000	0.75638	0.00321	0.75958
H 10	0.23978	0.00000	0.75709	0.00313	0.76022
C 11	-0.05453	1.99875	4.03892	0.01686	6.05453

C 12	-0.05573	1.99875	4.04014	0.01684	6.05573
C 13	-0.04915	1.99876	4.03349	0.01690	6.04915
C 14	-0.05820	1.99875	4.04267	0.01678	6.05820
C 15	-0.04997	1.99876	4.03431	0.01689	6.04997
C 16	-0.01400	1.99871	3.99744	0.01784	6.01400
C 17	-0.01400	1.99871	3.99743	0.01785	6.01400
C 18	-0.01419	1.99870	3.99769	0.01781	6.01419
C 19	-0.01396	1.99872	3.99737	0.01788	6.01396
C 20	-0.01417	1.99870	3.99766	0.01781	6.01417
C 21	-0.20364	1.99899	4.18824	0.01641	6.20364
C 22	-0.20523	1.99899	4.18998	0.01627	6.20523
C 23	-0.21068	1.99897	4.19525	0.01645	6.21068
C 24	-0.20019	1.99900	4.18492	0.01627	6.20019
C 25	-0.21139	1.99897	4.19606	0.01636	6.21139
H 26	0.23962	0.00000	0.75718	0.00319	0.76038
H 27	0.24025	0.00000	0.75662	0.00313	0.75975
H 28	0.24008	0.00000	0.75669	0.00323	0.75992
H 29	0.23968	0.00000	0.75718	0.00313	0.76032
H 30	0.24046	0.00000	0.75635	0.00319	0.75954
* Total *	0.00000	39.97718	89.65406	0.36876	130.00000

EDA Analysis of Sandwich-Type Aggregates

The bonding between bowl-shaped fragments and positively charged alkali metal belt (see Figures S12 and S13 for details) was further investigated by the energy decomposition analysis (EDA) developed by Morokuma and by Ziegler and Rauk.[17] For this purpose, single-point calculations were performed by the ADF program package[18] with the same functional. All atoms were described by uncontracted Slater-type orbitals (STOs) with TZ2P quality as basis functions.[19] An auxiliary set of s, p, d, and f STOs was used to fit the molecular densities and to represent the Coulomb and exchange potentials accurately in each SCF cycle.[20] Scalar relativistic effects have been taken into account by ZORA. Further details on the EDA can be found in literature.[21]

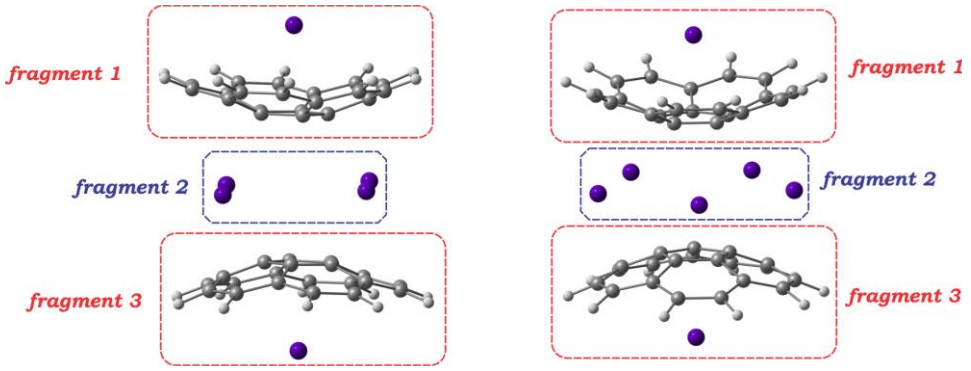

Figure S12. EDA Fragmentation scheme in **1**-*small* and **1H**-*small* models (*left*) as well as for **1**$^{4-}$-*small* (*right*).

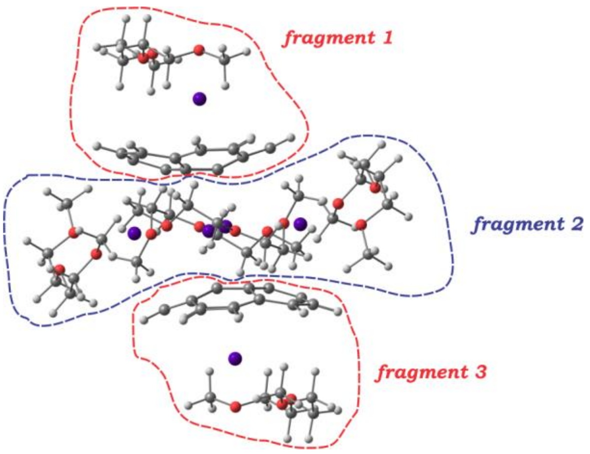

Figure S13. EDA Fragmentation scheme in **1**-*full* and **1H**-*full* models.

References

[1] N. V. Kozhemyakina, J. Nuss and M. Jansen, *Z. Anorg. Allg. Chem.*, 2009, **635**, 1355-1361.

[2] G. Mehta and G. Panda, *Tetrahedron Lett.*, 1997, **38**, 2145-2148.

[3] L. T. Scott, P.-C. Cheng, M. M. Hashemi, M. S. Bratcher, D. T. Meyer and H. B. Warren, *J. Am. Chem. Soc.*, 1997, **119**, 10963-10968.

[4] A. Sygula, G. Xu, Z. Marcinow and P. W. Rabideau, *Tetrahedron*, 2001, **57**, 3637-3644.

[5] (a) M. Baumgarten, J. L. Gherghel, M. Wagner, A. Weitz, M. Rabinovitz, P.-C. Cheng and L. T. Scott, *J. Am. Chem. Soc.*, 1995, **117**, 6254-6257. (b) G. Zilber, V. Rozenshtein, P.-C. Cheng, L. T. Scott, M. Rabinovitz and H. Levanon, *J. Am. Chem. Soc.*, 1995, **117**, 10720-10725.

[6] *SADABS, Bruker AXS.* **2001**.

[7] G. M. Sheldrick, *SHELXTL, Version 6.14, University of Göttingen, Germany* **2000**.

[8] A. A. Granovsky, Firefly, v.8.1.0, http://classic.chem.msu.su/gran/gamess/index.html.

[9] F. Neese, *ORCA*; University of Bonn: Bonn, Germany, **2009**.

[10] (a) D. A. Pantazis, X.-Y. Chen, C. R. Landis and F. Neese, *J. Chem. Theory Comput.*, 2008, **4**, 908-919. (b) M. Buhl, C. Reimann, D. A. Pantazis, T. Bredow and F. Neese, *J. Chem. Theory Comput.*, 2008, **4**, 1449-1459.

[11] F. Neese, F. Wennmohs, A. Hansen and U. Becker, *Chem. Phys.*, 2009, **356**, 98-109.

[12] (a) F. Weinhold and C. A. Landis, *Valency and Bonding: A Natural Bond Orbital Donor−Acceptor Perspective*, Cambridge University Press: Cambridge, 2005. (b) A. E. Reed, L. A. Curtiss and F. Weinhold, *Chem. Rev.*, 1988, **88**, 899-926.

[13] (a) E. D. Glendening, J. K. Badenhoop, A. E. Reed, J. E. Carpenter, J. A. Bohmann, C. M. Morales and F. Weinhold, *NBO 6.0*, 2013, University of Wisconsin, Madison. (b) E. D. Glendening, C. R. Landi and F. Weinhold, *J. Comp. Chem.*, 2013, **34**, 1429.

[14] K. Yamaguchi, Y. Takahara and T. Fueno *in*: V.H. Smith (Ed.) *Applied Quantum Chemistry*, Reidel, Dordrecht, 1986, pp 155.

[15] A. A. Granovsky, *J. Chem. Phys.*, 2011, **134**, 214113.

[16] H. Nakano, *J. Chem. Phys.*, 1993, **99**, 7983-7992.

[17] (a) K. J. Morokuma, *Chem. Phys.*, 1971, **55**, 1236. (b) T. Ziegler and A. Rauk, *Inorg. Chem.*, 1979, **18**, 1558. (c) T. Ziegler and A. Rauk, *Inorg. Chem.*, 1979, **18**, 1755.

[18] (a) C. Fonseca Guerra, J. G. Snijders, G. te Velde and E. J. Baerends, *Theor. Chem. Acc.*, 1998, **99**, 391. (b) *ADF2008.01*, SCM, Vrije Universiteit, Amsterdam,

Netherlands, http://www.scm.com. (c) G. te Velde, F. M. Bickelhaupt, E. J. Baerends, C. Fonseca Guerra, S. J. A. van Gisbergen, J. G. Snijders and T. Ziegler, *J. Comput. Chem.*, 2001, **22**, 931.

[19] J. G. Snijders, E. J. Baerends and P. Vernooijs, A*t. Data Nucl. Data Tables*, 1982, **26**, 483.

[20] J. Krijn and E. J. Baerends, *Fit Functions in the HFS-Method*, 1984, Internal Report (In Dutch), Vrije Universiteit, Amsterdam, Netherlands.

[21] (a) M. Lein and G. Frenking, in *Theory and Application of Computational Chemistry*, ed. Dekstra, C. 2005. (b) M. von Hopffgarten and G. Frenking, in *Computational Inorganic and Bioinorganic Chemistry*, ed. E. Solomon, 2009, pp. 3-15. (c) M. Lein, A. Szabo, A. Kovac and G. Frenking, *Faraday Discuss*., 2003, **124**, 365. (d) G. Frenking, K. Wichmann, N. Frohlich, C. Loschen, M. Lein, J. Frunzke and V. M. Rayon, *Coord. Chem. Rev.*, 2003, **238-239**, 55-82. (e) A. Krapp, F. M. Bickelhaupt and G. Frenking, *Chem. Eur. J*., 2006, **12**, 9196-9216. (f) A. Krapp and G. Frenking, *J. Comput. Chem*., 2007, **28**, 15-24. (g) M. von Hopffgarten and G. Frenking, *WIREs Comput. Mol. Sci*., 2012, **2**, 9196.
